# Synthesis and Characterization of Edaravone Analogues as Remyelinating Agents and Putative Mechanistic Probes

**DOI:** 10.3390/molecules28196928

**Published:** 2023-10-04

**Authors:** Eleonora Colombo, Stefania Olla, Cristina Minnelli, Alessia Formato, Caterina Veroni, Silvia Corbisiero, Mattia Pericolo, Chiara Siguri, Giovanna Mobbili, Cristina Agresti, Pierfausto Seneci

**Affiliations:** 1 Chemistry Department, University of Milan, Via Golgi 19, 20133 Milan, Italy; ecolombo2@bwh.harvard.edu (E.C.); mattia.pericolo12@gmail.com (M.P.); 2 Biomedical and Genetic Research Institute (IRGB, National Research Council (CNR)), University Campus, Monserrato, 09042 Monserrato, Italy; stefania.olla@irgb.cnr.it (S.O.); chiara.siguri@irgb.cnr.it (C.S.); 3 Department of Life and Environmental Sciences, Marche Polytechnic University, Via Brecce Bianche, 60131 Ancona, Italy; c.minnelli@staff.univpm.it (C.M.); g.mobbili@staff.univpm.it (G.M.); 4 Department of Neuroscience, National Institute of Health (ISS), Viale Regina Elena 299, 00161 Rome, Italy; a.formato92@gmail.com (A.F.); caterina.veroni@iss.it (C.V.); silvia.corbisiero98@gmail.com (S.C.)

**Keywords:** edaravone, amyotrophic lateral sclerosis, prodrugs, mechanism of action, chemical probes, antioxidant activity, remyelination, absorption-distribution-metabolism-excretion (ADME), medicinal chemistry

## Abstract

Edaravone (EDA), an antioxidant drug approved for the treatment of ischemic stroke and amyotrophic lateral sclerosis, was recently proposed as a remyelinating candidate for the treatment of multiple sclerosis. Here, we synthesized twelve EDA analogues **2b**–**4c** showing three substitution patterns **A**–**C**, searching for improved remyelinating agents and putative molecular targets responsible for their regenerative activity. We profiled them in three primary assays to determine their stimulation of oligodendrocyte progenitor cell metabolism (tetrazolium MTT assay), their antioxidant potential (2,2-diphenyl-1-picrylhydrazyl-DPPH assay) and to predict their bioavailability (virtual ADME profile). Active 4′-carboxylate **2b**, 4′-ester 2c and N^1^-carbamate-4′-ester **4a** were further characterized, justifying their in vitro effects and selecting **4a** as a putative EDA 1 prodrug suitable for in vivo testing.

## 1. Introduction

Multiple sclerosis (MS) is a chronic, inflammatory, demyelinating and neurodegenerative disease of the central nervous system (CNS) [1]. Current treatments for MS are effective in controlling the inflammatory component of the disease but fail in preventing the continuous loss of axons and neurons that leads to a permanent neurological disability [2]. The repair of damage to the myelin sheaths that surround axons and allow the propagation of nerve signals is inadequate in MS, particularly in the more advanced stages of the disease. The generation of new myelin-forming oligodendrocytes involves the differentiation of a population of glial cells, the oligodendrocyte progenitor cells (OPCs). The identification of drugs capable of inducing differentiation of OPCs present within damaged areas into remyelinating oligodendrocytes is an opportunity for MS treatment aimed to promote tissue repair [3].

**Figure 1:**
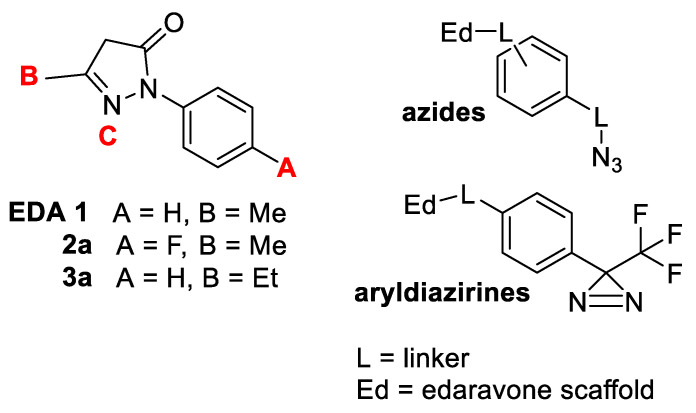
Left—EDA 1; remyelination-active **2a**, **3a**; A–C substitution patterns. Right—selected photoactivatable azide and diazirine probes.

We [4] and others [5,6] determined the remyelinating activity of the radical scavenger edaravone (EDA) **1a**, a neuroprotective agent for the treatment of ischemic stroke and/or amyotrophic lateral sclerosis (ALS) in Japan, USA and Canada [7]. We also identified two positions (**A** and **B**, Figure 1) whose limited substitutions (**2a** and **3a**, Figure 1) were compatible with remyelination in different in vitro and ex vivo disease models [4]. Here, we designed and assessed synthetic strategies to provide twelve EDA analogues, including seven **A**, two **B** and three **C** substitutions (respectively, **2b**–**h**, **3b**,**c** and **4a**–**c**, Figure 1); the C pattern was introduced to protect the redox-sensitive EDA nucleus and provide access to alcohol **2g** and azide **2h**. We also inserted four photoactivatable probes [8] (azides **2f**, **2g** and **2j**, and aryldiazirine **2h**, Figure 1) for covalent binding and mass spectrometry-proteomics identification of any unknown protein target responsible for remyelination.

We determined the biological activity and physicochemical properties of twelve EDA analogues in a primary biological/ADME profiling cascade, and further tested three active analogues to rationalize their in vitro activity, to select an early lead for future studies.

## 2. Results

### 2.1. Synthesis

**Scheme 1:**
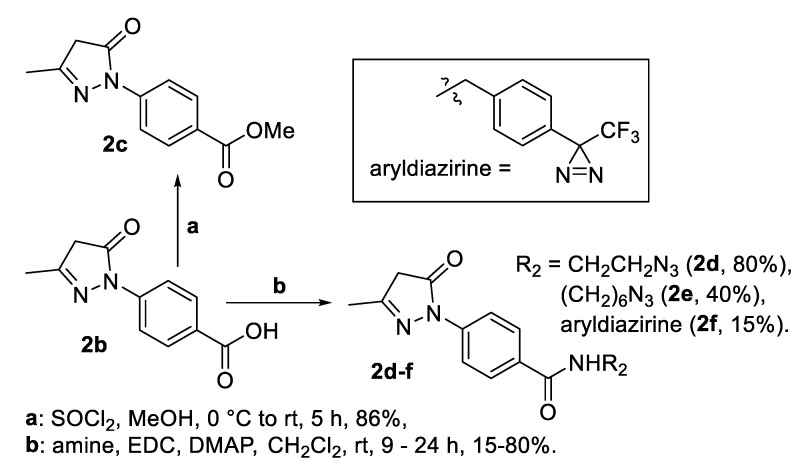
Synthesis of 4′-EDA ester **2c** and photoactivatable 4′-amide EDAs **2d**–**f**.

Esterification of commercial 4′-carboxy EDA **2b** [9] (step a, Scheme 1) led to 4′-methyl ester **2c**; attempts with other alcohols were unsuccessful, as were reduction attempts on ester **2c**, due to the well-known keto-enolic EDA tautomeric equilibrium [10].

We obtained 4′-EDA amides bearing azides **2d, 2e** and diazirine **2f** through amidation of carboxylate **2b** (step b, Scheme 1); the latter was obtained with a poor, unoptimized yield due to the limited diazirine stability.

**Scheme 2:**
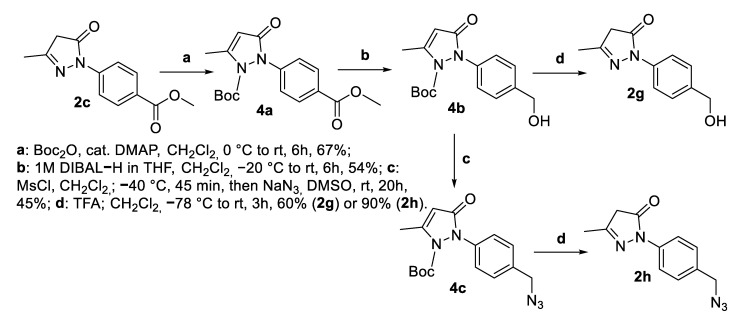
Synthesis of N^1^-carbamate EDA analogues **4a**–**c**, 4′-hydroxymethyl EDA **2g** and 4′-azidomethyl EDA **2h**.

Then, we targeted N^1^-protected carbamates **C** as gateways to hydroxymethyl alcohol **2g** and azide **2h** after N^1^-deprotection. N^1^-Boc 4′-EDA ester **4a** was obtained by protection of EDA 4′-ester **2c** (step a, Scheme 2). Ester 2c was reduced to N^1^-Boc 4′-hydroxymethyl **4b** in moderate yield (step b) and converted into N^1^-Boc 4′-azide EDA **4c** through a mesylation–substitution protocol (step c). N^1^-Boc **4b** and **4c** were deprotected in mild acidic conditions, respectively, to 4′-hydroxymethyl EDA **2g** and 4′-azidomethyl EDA **2h** (step d, Scheme 2).

**Scheme 3:**
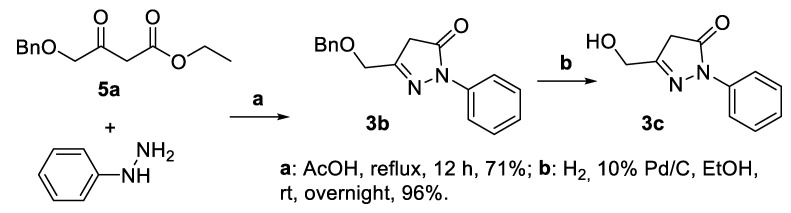
Synthesis of 5-substituted EDA analogues **3b**,**c**.

As for pattern **B**, we slightly modified a known [11] EDA synthesis by condensing phenylhydrazine with 3-oxobutanoate **5a** [12] to yield 5-benzyloxymethyl EDA **3b** (step a, Scheme 3); the latter was debenzylated (step b) to 5-hydroxymethyl EDA **3c**.

### 2.2. Primary Biological/ADME Profiling of EDA Analogues ***2b***–***4c***

**Figure 2:**
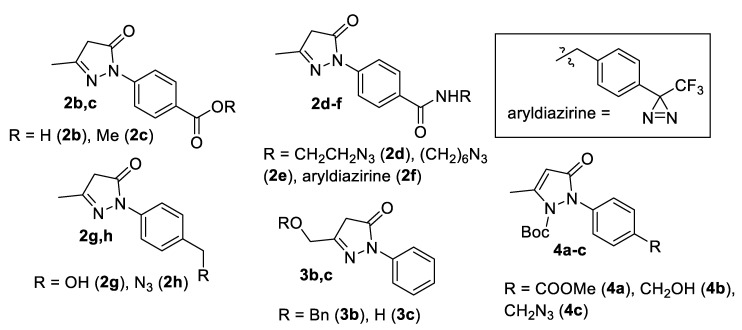
Structure of twelve EDA analogues **2b**–**4c** submitted to primary activity profiling.

Twelve EDA analogues (4′-carboxylate **2b**; 4’-ester **2c**; 4′-azides **2d, 2e** and **2h**; 4′-diazirine **2f**; 4′-alcohol **2g**; 5-hydroxymethyl **3b**; 5-azidomethyl **3c**; *N*^1^-carbamates **4a**–**c**, Figure 2) were tested in three primary assays.

Since the stimulatory effect of EDA **1** on OPC development results in an increase in their metabolism [4], EDA analogues were tested in a 3-(4,5-dimethylthiazol-2-yl)-2,5-diphenyltetrazolium bromide (MTT) enzymatic assay [13], and in an antioxidant–radical scavenging 2,2-diphenyl-1-picrylhydrazyl (DPPH) assay [14]. Such profiling could determine whether any EDA analogue shows either OPC prodifferentiating activity or antioxidant activity, or both. We also computationally predicted the adsorption, distribution, metabolism, and excretion (ADME) properties of EDA analogues to estimate their bioavailability.

**Figure 3:**
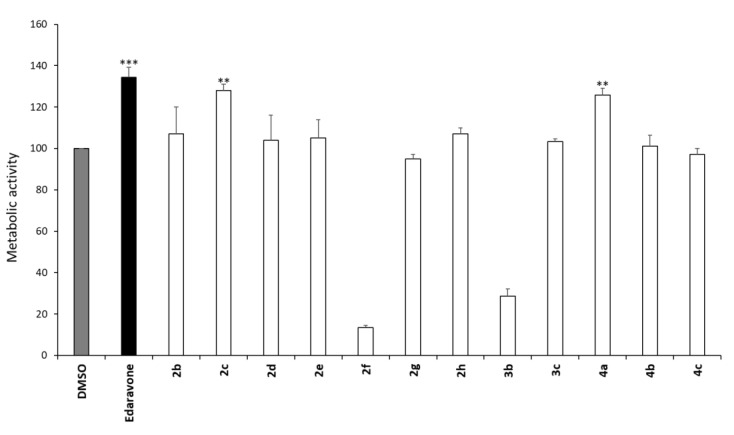
Effect on metabolic activity of OPCs by EDA 1 and its analogues **2b**–**4c** in the MTT assay (96-well plates, 48 h incubation). The histogram shows the rate of tetrazolium salt reduction into formazan. Values represent the mean ± SEM of three experiments run in triplicate (** = *p* ≤ 0.001; *** = *p* ≤ 0.0001).

Purified mouse OPCs were grown for 24 h and treated with EDA **1** and analogues **2b**–**4c** (10 μM) for 44 h; then, MTT was added and OPCs were incubated for 4 h. As shown in Figure 3, 4′-ester EDA **2c** (pattern **A**) and N^1^-carbamate EDA 4′-ester **4a** (pattern C) stimulated OPC metabolism comparably to EDA **1**. EDA aryldiazirine **2f** and alcohol **3a** were cytotoxic at this concentration and inactive at lower dosages (not shown). Other EDA analogues, including putative photoactivatable azides **2d, 2e** and **2h**, did not stimulate OPC development compared to DMSO controls.

**Figure 4:**
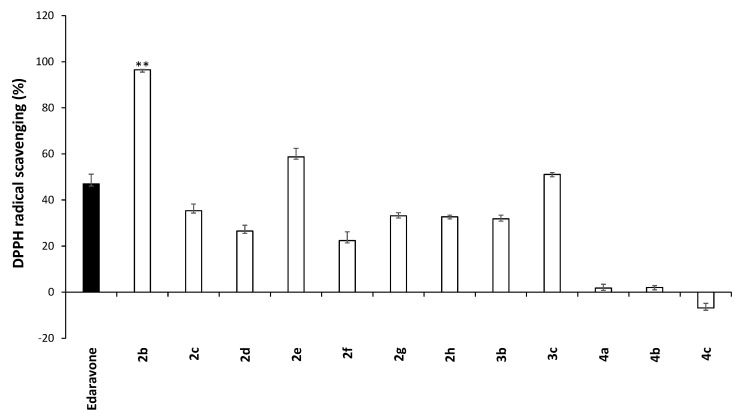
Radical scavenging/antioxidant activity of EDA **1** and its analogues **2b**–**4c** in the DPPH assay (30 min incubation, MeOH). Values represent the mean ± SEM of three experiments run in triplicate (** = *p* ≤ 0.001).

A DPPH assay was carried out after 30 min of incubation with EDA **1** and its analogues in reported conditions [14] to determine their radical scavenging ability. Results, expressed as % inhibition, are shown in Figure 4.

We observed limited antioxidant activity, with 4′-carboxy EDA **2b** being significantly more potent than EDA **1**; we hypothesize that the moderately electron-donating effect of carboxylate in **2b**, due to its negative charge at physiological pH, could better stabilize the radical formed after hydrogen donation to DPPH. Most **A**–**B** analogues showed comparable antioxidant activity to EDA **1** (ester **2c**, azides **2d** and **h**, alcohols **2g** and **3b**, and ether **3a**). N^1^-Boc EDAs **4a**–**c** (**C**) could not act as radical scavengers because a tautomeric EDA equilibrium [10] is prevented by their substitutions.

We used the QikProp tool [15] to predict the ADME properties of EDA analogues **1**–**4c**; the full panel is provided in the Supplementary Materials (Table S1). Here, we focus on lipophilicity and cell permeability in the CNS, showing the partition between n-octanol and water (LogP), intestinal absorption (Caco-2 permeability), partition between blood and brain (LogBB), CNS absorption (Madin–Darby canine kidney—MDCK permeability) and predicted CNS access. These values are reported in Table 1, columns 2 to 6, with a general “druggability” indication (Lipinski Rule of Five violations, column 7).

EDA **1** was predicted as permeable in Caco-2 (oral) and MDCK (blood–brain barrier-BBB) models; its LogP and LogBB values indicated an acceptable lipo- vs. hydrophilicity ratio. MTT-active 4′-ester **2c** and N^1^-Boc 4′-ester EDA **4a** were characterized as orally bioavailable, with limited CNS access. Conversely, poor bioavailability was predicted for strong antioxidant carboxylate **2b**; photoactivatable azides **2f**, **2g** and **2j**; and diazirine **2h**. EDA analogues **2b**–**4c** showed no major violations of Lipinski’s Rule of Five (molecular weight, rotatable bonds, hydrogen bond donor and acceptor groups).

### 2.3. Detailed Characterization—EDA Analogues ***2b***, ***2c***, ***4a***

Primary profiling identified three active EDA analogues, none endowed with both OPC stimulation and antioxidant properties as EDA **1**. We focused on strong antioxidant 4-carboxy EDA **2b** (DPPH assay) on moderately remyelinating 4′-ester EDA **2c** and N^1^-Boc 4′-ester EDA **4a** (MTT assay) to better characterize their biological activity. EDA **1** was also tested, as a positive standard.

We experimentally confirmed their predicted poor to medium–good permeability profile by measuring their LogD at physiological pH through a known procedure [14], and by determining their percentage bound to model lipid bilayers after incubation, as a measure of their affinity for cell-membrane-like liposomes. Results are shown in Table 2 (LogD_7_._4_, column 2; interaction with liposomes at 20 °C and 37 °C, columns 3 and 4, respectively).

N^1^-Boc 4′-ester **4a** showed a better LogD_7_._4_ value than EDA **1** and 4′-ester EDA **2c** (column 2); 4′-carboxy EDA **2b** showed poor affinity for lipophilic environments. A similar pattern was observed with pre-formed liposomes at 20 °C, showing strong permeation/partition into liposomes for lipophilic **4a**, medium for 4′-ester **2c** and moderate for EDA **1** and 4′-carboxy EDA **2b** (column 3); partition into liposomes increased with temperature (column 4, 37 °C), with the exception of N^1^-carbamate 4′-ester EDA **4a**.

Density functional theory (DFT) methods were used to rationalize the experimental antioxidant activity of EDA analogues using Jaguar^TM^ software [16], and optimizing structures at the 6–311++G(d,p) level for all atoms, as described in detail in the Material and Methods section. Predicted Molecular Electrostatic Potentials (MEPs) and Average Local Ionization Energies (ALIEs) are shown in the Supplementary Materials (Figure S1) and tabulated as energy values in Table S2.

The HOMO (Highest Occupied Molecular Orbitals) and LUMO (Lowest Unoccupied Molecular Orbitals) representation, and the ΔE energy gap for EDA analogues are tabulated in Table 3 (second to fourth column, respectively); their images are shown in the Supplementary Materials (Figure S2).

The HOMO–LUMO ΔEs calculated for EDA **1** and analogues **2b**, **2c** and **4a** predicted a trend to donate electrons, as their ionization potential (IP = −E_HOMO_, fifth column, Table 3) is higher than their electron affinity (EA = −E_LUMO_, sixth column). 4′-Carboxy EDA **2b** and its ester **2c** exhibited the lowest ΔEs, predicting higher antioxidant potency [17]; these and N^1^-carbamate 4′-ester EDA **4a** showed the highest EAs (sixth column), hinting to single electron transfer reactions (hydrogen free radical donation). HOMOs were delocalized on the phenyl and pyrazolone carbon atoms, while LUMOs were delocalized to the phenyl ring except for EDA 1, whose LUMO was delocalized to the pyrazolone ring (Figure S2).

### 2.4. Stability Studies—EDA Analogues ***2c*** and ***4a***

**Figure 5:**
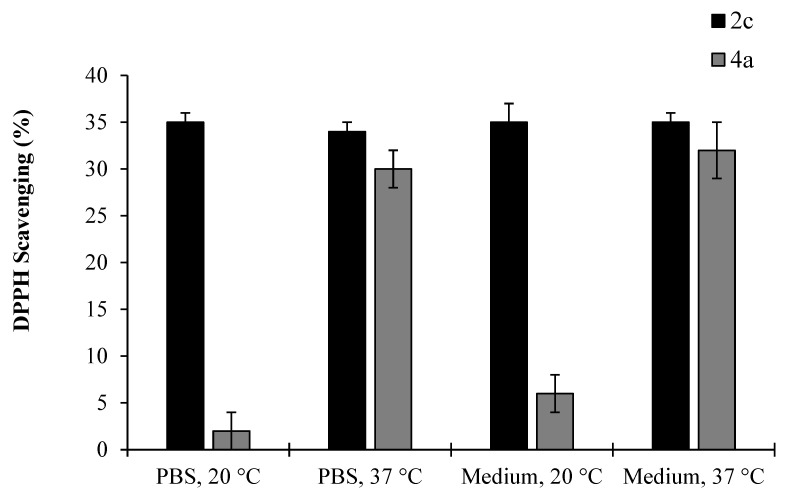
DPPH radical scavenging activity of 4′-ester EDA **2c** and N^1^-carbamate 4′-ester EDA **4a** after incubation in biological media at 20 °C and 37 °C.

We investigated the stability of 4′-ester EDA **2c** and N^1^-carbamate 4′-ester EDA **4a** (respectively, black and grey bars, Figure 5) in biological media, checking both ester and N^1^-carbamate stability in **4a** both by thin-layer chromatography (TLC) and by checking the DPPH radical scavenging activity of **2c** and **4a** after incubation.

Namely, **2c** and **4a** were incubated in PBS and in standard medium at 20 °C and 37 °C for 20 min; then, DPPH was added and the radical scavenging activity was measured in all media [14]. The expected radical scavenging activity by 4′-ester EDA **2c** was observed (dark bars, Figure 5), and its stability in biological media was confirmed by TLC. DPPH-inactive N^1^-carbamate 4′-ester EDA **4a** did not scavenge DPPH in both media at 20 °C (first and third light bars), and its stability was confirmed by TLC. Conversely, significant antioxidant activity was detected at 37 °C (second and last light bars, Figure 5) due to loss of t-Boc and conversion into DPPH-active 4′-ester EDA **2c**; TLC confirmed N^1^-carbamate hydrolysis and **2c** formation at 37 °C.

## 3. Discussion and Conclusions

Potent, safe myelin-repair-promoting treatments could be an effective therapy for MS and, possibly, for other demyelinating diseases [18]. EDA **1** is a promising scaffold to build potent and selective remyelinating agents through a structural optimization program; importantly, its repurposing to treat MS could rapidly provide, after clinical evaluation, a well-tolerated and effective drug [4,5,6].

Twelve EDA analogues **2b**–**4c** were synthesized, and small substituents were introduced on **A**–**C** substitution patterns (Figure 2). Most of them were predicted by computational methods to be drug-like, BBB-permeable compounds.

Poorly bioavailable 4′-carboxy EDA **2b** showed significantly higher antioxidant activity than parent EDA **1**, while lipophilic N-aryl- and 5-substituted EDA analogues (ester **2c**, azides **2d** and **2h**, alcohols **2g** and **3a**, and ether **3b**) were comparable to EDA **1**. We confirm a known, positive effect of lipophilicity on radical scavenging, and the compatibility of N^2^ aryl substitutions with antioxidant activity [19]. Unfortunately, lack of activity on OPCs prevented the use of putative photoactivatable EDA azides **2d**, **2e** and **2h**, and diazirine **2f** as tools for drug-target identification.

4′-Ester EDA **2c** and N^1^-Boc 4′-ester EDA **4a** showed comparable stimulation of OPC metabolism, and possibly of remyelination, to EDA **1**. The activity for **4a** was particularly intriguing, as N^1^-functionalization of EDA **1** prevents tautomeric equilibria and should cancel antioxidant activity.

Primary profiling could not exclude the antioxidant effects of EDA **1** being responsible for OPC prodifferentiating/remyelinating activity in cells. ADME profiling indicated that 4′-ester EDA **2c** is bioavailable and should be hydrolyzed in vitro/in vivo by esterases to the potent antioxidant 4′-acid ETA **2b**, possibly leading to remyelination. We hypothesize that at least part of the OPC stimulatory activity of 4′-ester **2c** in the MTT assay could be due to in situ hydrolysis to 4′-acid ETA **2b**; conversely, the poor bioavailability of **2b** may preclude its access to the intracellular space, thus preventing OPC stimulation and remyelination after its direct in vitro/in vivo administration.

The metabolic OPC stimulation–remyelinating efficacy of DPPH-inactive N^1^-carbamate 4′-ester EDA **4a** was surprising, as was its decreased permeation into lipophilic liposomes at 37 °C, until we observed the instability of its N^1^-carbamate, which was fully removed after 20 min’ incubation at 37 °C in either PBS or standard culture medium. We suggest that 4a may act as a prodrug of 4′-ester EDA **2c** in suitable conditions; we also suggest that both 4′-ester EDA **2c** and N^1^-carbamate 4′-ester EDA **4a** may act as prodrugs of the potent antioxidant, poorly bioavailable 4′-carboxy EDA **2b**, through esterase-promoted ester hydrolysis. Please note that a long, 48 h incubation time at room temperature (rt) in the MTT assay should allow both **2c** and **4a** to be converted into 4′-acid EDA **2b** in OPCs and that a short 30 min incubation at rt in the DPPH assay could not lead to carbamate hydrolysis for DPPH-inactive **4a**, while raising the temperature at 37 °C enabled the formation of DPPH-active **2c**. Consequently, the conversion of N^1^-carbamate 4′-ester **4a** into 4′-ester **2c** at 37 °C in the liposomal interaction explains the observed reduction in liposomal affinity/permeability vs. testing at 20 °C (no N^1^-carbamate hydrolysis). Our efforts to validate this prodrug hypothesis, and to modulate the structure and the stability of N^1^-EDA carbamates **4** through other substitutions, will be reported in due time.

The identification of a molecular target responsible for the remyelinating activity of EDA **1** through synthetic efforts could not be accomplished due to the lack of evidence for an alternative mechanism of action with respect to radical scavenging. Further studies, focused on biology-centered approaches to target identification and validation, are currently ongoing and will be reported in due time.

## 4. Materials and Methods

### 4.1. Synthesis

#### 4.1.1. General

Chemical reactions were carried out in oven-dried glassware, using dry solvents under a nitrogen atmosphere. Dry and normal solvents were purchased (Sigma-Aldrich SRL., Milan, Italy) and directly used in chemical reactions. Commercial reagents were purchased from Sigma-Aldrich, and analytically examined for integrity before handling. Direct phase flash chromatography columns for products’ purification were filled with commercial silica gel (240–400 mesh, Merck, Darmstadt, Germany). Thin-layer chromatography (TLC) was employed to monitor reactions using Merck-precoated 60F_254_ silica gel plates. UV light at 254 nm was used as a detection method for chromophore-bearing compounds; alternatively, plate charring was carried out either with 50% aqueous H_2_SO_4_ or 1% aqueous permanganate. ^1^H- and ^13^C-nuclear magnetic resonance (NMR) spectra were recorded on Bruker DRX-400 and Bruker DRX-300 instruments in the most appropriate deuterated solvent (CDCl_3_, CD_3_OD or DMSO-d_6_) depending on the solubility of intermediates and target compounds. Tetramethylsilane (TMS) was used as an internal standard to report chemical shift values (δ) for proton and carbon signals, expressed in parts per million (ppm). Electrospray ionization (ESI) mass spectrometry (MS) spectra were recorded on a Waters Micromass Q-Tof micro mass apparatus, while high-resolution (HR)-ESI MS spectra were recorded on an FT-ICR APEX_II_ spectrometer (Bruker Daltonics, Billerica, MA, USA).

4′-Carboxy EDA **2b** was purchased (Sigma-Aldrich SRL., Milan, Italy) and used as such. The synthesis of ETA ester **2c** and N^1^-carbamate ester **4a** are described here. The synthesis of any other EDA analogue is provided in the Supporting Information. NMR spectra and proton assignments are reported for EDA analogues **2c**–**4c** in the Supplementary Materials.

#### 4.1.2. Synthesis of Methyl 4-(3-methyl-5-oxo-4,5-dihydro-1H-pyrazol-1-yl)benzoate **2c**

Thionyl chloride (0.99 mL, 14.0 mmol) was added to a stirred solution of carboxylate **2b** (500 mg, 2.34 mmol) in MeOH (30 mL) at 0 °C. The reaction was left stirring at rt for 5 h. Reaction monitoring (TLC, eluent mixture: 7:3 n-hexane/AcOEt) showed the formation of a reaction product and the disappearance of **2**. The solvent was then evaporated under reduced pressure, and the crude was dissolved in CH_2_Cl_2_ (15 mL). Saturated aqueous NaHCO_3_ (15 mL) was added to the organic layer, and the aqueous layer was extracted with CH_2_Cl_2_ (3 × 15 mL). The organic extracts were dried with Na_2_SO_4_ and evaporated under reduced pressure to obtain pure **2c** (459 mg, 2.01 mmol, 86% yield) as a white solid. Analytical characterization—**2c***:* **^1^H-NMR** (CDCl_3_, 400 MHz): δ(ppm) = 8.05 (d, 2H, *J* = 9.0 Hz), 7.98 (d, 2H, *J* = 9.0 Hz), 3.90 (s, 3H), 3.45 (s, 2H), 2.20 (s, 3H). **^13^C-NMR** (CDCl_3_, 101 MHz): δ(ppm) 170.8, 166.6, 157.0, 141.8, 130.5 (2C), 126.0, 117.6 (2C), 52.0, 43.1, 17.0. **MS (ESI^+^)**, m/z: calculated for C_12_H_12_N_2_O_3_ MW 232.08, found 233.33 (M + H^+^).

#### 4.1.3. Synthesis of Tert-butyl 2-(4-(methoxycarbonyl)phenyl)-5-methyl-3-oxo-2,3-dihydro-1H-pyrazole-1-carboxylate **4a**

A solution of di-tert-butyl dicarbonate (197 mg, 0.91 mmol) in dry CH_2_Cl_2_ (2.5 mL) was added under nitrogen atmosphere to a stirred solution of ester **2c** (150 mg, 0.646 mmol) in dry CH_2_Cl_2_ (2.5 mL) at 0 °C. Then, a solution of DMAP (0.7 mg, 0.0646 mmol) in dry CH_2_Cl_2_ (1 mL) was added at 0 °C, and the reaction mixture was gently warmed and stirred at rt for 6 h. The reaction was monitored with TLC (eluent mixture 8:2 n-hexane/AcOEt) until disappearance of **2c**. The reaction mixture was washed with saturated aq. NH_4_Cl (7 mL). The organic extracts were dried with Na_2_SO_4_ and evaporated under reduced pressure to a crude, which was purified with flash chromatography (silica gel, eluent mixture 8:2 n-hexane/AcOEt). Pure title compound **4a** was obtained as a white solid (79.2 mg, 0.238 mol, 67% yield). Analytical characterization—**4a**: **^1^H-NMR** (CDCl_3_, 400 MHz): δ(ppm) 8.10 (d, 2H, *J* = 8.8 Hz), 7.70 (d, 2H, *J* = 8.8 Hz), 6.09 (s, 1H), 3.91 (s, 3H), 2.30 (s, 3H), 1.46 (s, 9H). **^13^C-NMR** (CDCl_3_, 101 MHz): δ(ppm) 166.5, 149.8, 148.7, 145.4, 141.9, 130.8 (2C), 128.1, 121.6 (2C), 96.4, 85.7, 52.3, 27.6 (3C), 14.6. **MS (ESI^+^)**, m/z: calculated for C_17_H_20_N_2_O_5_ MW 332.14, found 333.26 (M + H^+^).

### 4.2. Biology

#### 4.2.1. Animals

CD1 Swiss mice were purchased from Harlan Laboratories (San Pietro Al Natisone, Udine, Italy). All experimental procedures involving the use of CD1 Swiss mice were conducted in accordance with Directive 2010/63/EU of the European Parliament and of the Council concerning the protection of animals used for scientific purposes. The experiments received official authorization from the “Istituto Superiore di Sanità”—Service for Biotechnology and Animal Welfare and were also approved by the Italian Ministry of Health (Authorization n. 87/2017-PR—09/23/2018).

#### 4.2.2. OPC Cultures

Primary cultures of purified OPC (>99%) were obtained from the cerebral hemispheres of newborn CD1 Swiss mice [4] according to the authorization for the care and use of laboratory animals mentioned in Section 4.2.1. OPCs were grown in a defined serum-free Dulbecco’s Modified Eagle Medium (DMEM) without thyroid hormones at 1 × 10^5^ cells/cm^2^ into poly-L-lysine-coated 96-well microtiter plates for 3 days.

#### 4.2.3. 3-(4,5-Dimethylthiazol-2-yl)-2,5-Diphenyltetrazolium Bromide (MTT) Reduction Assay

One day after plating, purified OPCs were incubated with or without EDA analogues (10 µM) in DMSO (0.001% vehicle) for 48 h. MTT reduction to formazan was carried out as previously described [4]. MTT (Sigma-Aldrich, Milan, Italy) was added to the culture medium at a final 0.25 mg/mL concentration during the last 4 h of incubation.

#### 4.2.4. 2,2-Diphenyl-1-picrylhydrazyl (DPPH) Radical Scavenging Assay 

The DPPH free radical scavenging activity of EDA analogues was determined using a previously described method [14]. Appropriate aliquots of putative antioxidants **1–13c** were mixed with DPPH in methanol (25 µM EDA analogue, 100 µM DPPH, final concentration); the reaction mixtures were shaken and then incubated for 30 min in the dark. Their absorbances, and the absorbance of a DPPH solution as a negative control, were then measured at 517 nm with a BioTek SynergyHT MicroPlate Reader spectrophotometer (Marshall Scientific, Hampton, NH, USA) against methanol as blank. The scavenging percentage of each EDA analogue was calculated according to the following equation:

*DPPH radical scavenging (%) = [(A_control_—A_sample_)/A_control_]* × 100, (1)

where *A_control_* is the control absorbance obtained by adding a methanol aliquot equal to the EDA analogue solution volume added to the methanolic DPPH solution and *A_sample_* is the absorbance of each test solution after 30 min incubation. All experiments were repeated at least three times, and measurements were run in triplicate.

#### 4.2.5. Chemical Stability—DPPH Radical Scavenging Assay

4′-Ester **2c** and N^1^-carbamate ester **4a** were incubated in PBS and in a standard cell culture medium supplemented with FBS 10% at 20 °C and 37 °C. After 20 min, appropriate aliquots of the solutions were mixed with DPPH in methanol (25 µM, DPPH 100 µM, final concentration) and the DPPH radical scavenging assay was carried out as described above. Assay media after incubation were also monitored by TLC (eluent mixture: 1:1 cyclohexane/AcOEt).

#### 4.2.6. Determination of Distribution Coefficient (Log D_7_._4_)

LogD_7_._4_ values were determined by slightly modifying a reported procedure [20]. Namely, an 80 mM solution of each selected EDA analogue in DMSO was diluted in 1-octanol at a final 800 μM concentration. UV–vis spectra were acquired to determine λmax and the necessary dilutions to obtain absorption values for each tested analogue; between 0.5 and 1. 5 mL of each solution were placed in 15 mL conical centrifuge tubes, PBS was added (5 mL, pH 7.4) and the biphasic system was vigorously mixed using a vortex mixer (VELP Scientifica, Usmate Velate, MB, Italy) for 4 min. After centrifugation (2000× *g*, 30 min), the 1-octanol phase absorption was measured at each compound’ λmax; the procedure was repeated to acquire data in triplicate. The distribution coefficient *logD_7_._4_* was calculated according to the following equation:

*LogD_7_._4_ = Log ([C]_oct_/[C]_PBS_) = Log (A_f_/A_i_ − A_f_)* (2)

where *A_i_* is the pre-extraction measured absorbance in 1-octanol and *A_f_* is the post-extraction final measured absorbance in 1-octanol.

#### 4.2.7. Interaction Studies with Liposome Cell Membrane Models

EDA analogues dissolved in MeOH (0.1 mL) were added to 0.9 mL of large unilamellar phosphatidylcholine vesicles (PC LUVs) in PBS (3 mM lipid final concentration; 0.6 mM EDA analogue final concentration) and incubated at 37 °C for 20 min. Then, any remaining free EDA analogue was separated from liposomes by size-exclusion chromatography.

A disposable syringe (2.5 mL) packed with hydrated Sephadex G-50 resin was placed in a 15 mL plastic test tube and preconditioned with PBS; then, the liposomal suspension (1 mL) was gently added on the top of the syringe and centrifuged at 500× *g* for 10 min. The eluate was collected at the bottom of the test tube and lysed by addition of Triton X-100 (final concentration 1% *v/v*). The concentration of each EDA analogue in each solution was estimated with the Folin–Ciocalteu assay [21]. Namely, 50% Folin–Ciocalteu reagent (150 μL) was added to test samples (50 μL) into a 96-well microplate and shaken. After 10 min, a 7.5% sodium carbonate solution (0.1 mL) was added; the mixtures were incubated in the dark for 1 h at rt; and, finally, the absorbance was measured at 765 nm on a BioTek SynergyHT MicroPlate Reader spectrophotometer (Marshall Scientific, Hampton, NH, USA) using an appropriate blank. Reference calibration curves were plotted for every sample. The ratio (%) of the drug incorporated into the lipid bilayer was then calculated according to the following equation:

*% ratio of incorporated antioxidant = 100 × [incorporated amount]/[added amount]* (3)

Experiments were repeated at least three times, and measurements were run in triplicate.

### 4.3. Computational Studies

#### 4.3.1. In Silico ADME Predictions

The Schrodinger QikProp^TM^ tool (Small-Molecule Drug Discovery Suite 2021–1, Schrodinger, LLC, New York, NY, USA) was used to forecast the absorption, distribution, metabolism and excretion (ADME) profile of the compounds under investigation. This tool employs an algorithm that relies on establishing a correlation between experimentally determined properties and Monte Carlo statistical mechanics simulations involving organic solutes placed within a periodic box containing explicit water molecules. QikProp exploits multiple indicators to gauge the putative central nervous system (CNS) activity of a small molecule, thereby assessing its ability to access the brain compartment by crossing the blood–brain barrier (BBB). The main indicators include the following: (i) the LogBB, i.e., the blood–brain partition coefficient; (ii) the apparent Madin–Darby canine kidney (MDCK) permeability, i.e., forecasting a small molecule’s ability to permeate an MDCK layer, measured in nanometers per second (nm/sec); (iii) a CNS activity predictor, guessing the likelihood of a given compound to be CNS-active; and (iv) the apparent permeability through human colon carcinoma Caco-2 cell lines for compounds **2b**–**4c**, estimated so as to predict oral bioavailability and assess drug efflux. Finally, violations of Lipinski’s Rule of 5 (≤500 Daltons (Da) as molecular, ≤5 hydrogen bond donors, ≤10 hydrogen bond acceptors and LogP values ranging from 0 to 5) were recorded.

#### 4.3.2. Quantum Chemical Simulations

The Jaguar^TM^ tool (Small-Molecule Drug Discovery Suite 2021–1, Schrodinger, LLC, New York, NY, USA) was employed for electronic calculations such as Molecular Electrostatic Potential (MEP), Average Local Ionization Energy (ALIE), HOMO (Highest Occupied Molecular Orbital) and LUMO (Lowest Unoccupied Molecular Orbital) energies (and their energy gap (ΔE)) using DFT methods. Theoretical calculations were performed using B3LYP (Becke’s three-parameter exchange potential and the Lee–Yang–Parr correlation function) [22] and the 6–311++G(d,p) basis set. The PCM continuum model (water) was used for solvent effect prediction [23]. EDA analogues were initially submitted to a complete conformational and tautomeric search using quantum mechanics (QM) methods, and the lowest energy conformers were subsequently optimized with DFT. The electronic structures along with spin density distributions of HOMO and LUMO orbitals were presented in color-coded surfaces that give the location of positive and negative electrostatic potentials.

## Figures and Tables

**Table 1:** In silico CNS bioavailability parameters for EDA **1** and analogues **2b**–**4c**.

Compound ^a^	LogP, n-octanol/Water Ratio	Apparent Caco-2 Permeability nm/sec	Log BB, Brain/Blood Ratio	Apparent MDCK Permeability nm/sec	Predicted CNS Access ^b^	Lipinski Rule of 5 Violations
	1 to 3 good	<25 poor, >500 good	−2.7 to −1.5 good	<25 poor, >500 good	−2 to +2 ^b^	<2 good
**1** (MTT, DPPH)	1.320	2693	0.065	1443	1	0
**2b** (DPPH)	1.091	62	−0.974	31	−1	0
**2c** (MTT)	1.151	791	−0.561	384	0	0
**2d**	0.260	95	−1.815	38	−2	0
**2e**	1.634	91	−2.292	37	−2	0
**2f**	3.172	217	−1.261	376	−2	0
**2g**	1.063	761	−0.591	368	0	0
**2h**	0.319	224	−1.174	98	−2	0
**4a** (MTT)	2.227	726	−0.691	350	0	0
**4b**	2.167	664	−0.770	318	−1	0
**4c**	1.416	194	−1.383	84	−2	0
**3b**	3.184	2568	−0.293	1371	0	0
**3c**	0.800	838	−0.511	408	0	0

^a^ Brackets specify activity in MTT and/or DPPH assays. ^b^ +2, very likely to access its site of action in the CNS; +1, likely; 0, 50% likely; −1, unlikely; −2, unable to access the CNS.

**Table 2:** In vitro profiling for EDA **1** and selected analogues **2b**, **2c** and **4a**.

Compound	Log D_7_._4_^a^	Affinity for a Model Lipid Bilayer ^b^		Cu-Induced ROS Inhibition ^c,a^
		20 °C	37 °C	
**1**	1.1 ± 0.1	37 ± 3	53 ± 2	14 ± 2
**2b**	−0.74 ± 0.19	38 ± 4	45 ± 2	38 ± 5
**2c**	1.0 ± 0.1	52 ± 2	71 ± 2	53 ± 7
**4a**	2.3 ± 0.4	88 ± 6	77 ± 4	65 ± 6

^a^ ± standard deviation (SD); ^b^ % ratio of liposome-bound EDA analogue; ^c^ IC_50_, μM.

**Table 3:** Predicted HOMO, LUMO, ΔE, IP and EA values for EDA **1** and selected analogues **2b**, **2c** and **4a**.

Compound	HOMO (eV)	LUMO (eV)	∆E (eV)	IP (eV)	EA (eV)
**1**	−6.13	−1.01	5.12	6.13	1.01
**2b**	−6.34	−1.71	4.63	6.34	1.71
**2c**	−6.31	−1.66	4.65	6.31	1.66
**4a**	−6.45	−1.71	4.74	6.45	1.71

## Data Availability

Not applicable.

## Supplementary Materials

The following Supporting Information can be downloaded at: https://www.mdpi.com/article/10.3390/molecules28196928/s1: a Word file containing the detailed synthetic procedures for intermediate **5a** and EDA analogues **2d**–**h**, **3b**,**c** and **4b**,**c**; the full analytical characterization of EDA analogues **2c**–**4c**; the MEP and ALIE surfaces (Figure S1); the HOMO and LUMO representation (Figure S2) and energy values (Table S2) calculated through DFT analysis. References [24,25,26,27] are cited in the supplementary materials. The full ADME prediction set for EDA analogues **1**–**4c** (Table S1) is provided as a separate Excel file.

## Sample Availability

Not available.

## References

[1] McGinley M.P., Goldschmidt C.H., Rae-Grant A.D. (2021). Diagnosis and treatment of multiple sclerosis. JAMA.

[2] Goldschmidt C.H., McGinley M.P. (2021). Advances in the treatment of multiple sclerosis. Neurol. Clin..

[3] Tepavčević V., Lubetzki C. (2022). Oligodendrocyte progenitor cell recruitment and remyelination in multiple sclerosis: The more, the merrier?. Brain.

[4] Eleuteri C., Olla S., Veroni C., Umeton R., Mechelli R., Romano S., Buscarinu M., Ferrari F., Calò G., Ristori G. (2017). A Staged Screening of Registered Drugs Highlights Remyelinating Drug Candidates for Clinical Trials. Sci. Rep..

[5] Luo W., Xu H., Xu L., Jiang W., Chen C., Chang Y., Liu C., Tian Z., Qiu X., Xie C. (2023). Remyelination in neuromyelitis optica spectrum disorder is promoted by edaravone through mTORC1 signaling activation. Glia.

[6] Bakhtiari M., Ghasemi N., Salehi H., Amirpour N., Kazemi M., Mardani M. (2021). Evaluation of Edaravone effects on the differentiation of human adipose derived stem cells into oligodendrocyte cells in multiple sclerosis disease in rats. Life Sci..

[7] Cho H., Shukla S. (2021). Role of Edaravone as a Treatment Option for Patients with Amyotrophic Lateral Sclerosis. Pharmaceuticals.

[8] Ziegler S., Pries V., Hedberg C., Waldmann H. (2013). Target Identification for Small Bioactive Molecules: Finding the Needle in the Haystack. Angew. Chem. Int. Ed..

[9] Kumar B., Verma R.K. (1984). Esterification at Room Temperature: A Mixing Affair Only. Synth. Commun..

[10] Queiroz A.N., Martins C.C., Santos K.L.B., Carvalho E.S., Owiti A.O., Oliveira K.R.M., Herculano A.M., da Silva A.B.F., Borges R.S. (2020). Experimental and Theoretical Study on Structure-Tautomerism among Edaravone, Isoxazolone, and Their Heterocycles Derivatives as Antioxidants. Saudi Pharm. J..

[11] Xu Y., Smith R., Vivoli M., Ema M., Goos N., Gehrke S., Harmer N.J., Wagner G.K. (2017). Covalent inhibitors of LgtC: A blueprint for the discovery of non-substrate-like inhibitors for bacterial glycosyltransferases. Bioorg. Med. Chem..

[12] Jiang J., Sigua L.H., Chan A., Kalra P., Pomerantz W.C.K., Schönbrunn E., Qi J., Georg G.I. (2022). Dihydropyridine lactam analogs targeting BET bromodomains. ChemMedChem.

[13] Ghasemi M., Turnbull T., Sebastian S., Kempson I. (2021). The MTT Assay: Utility, Limitations, Pitfalls, and Interpretation in Bulk and Single-Cell Analysis. Int. J. Mol. Sci..

[14] Minnelli C., Laudadio E., Galeazzi R., Rusciano D., Armeni T., Stipa P., Cantarini M., Mobbili G. (2019). Synthesis, Characterization and Antioxidant Properties of a New Lipophilic Derivative of Edaravone. Antioxidants.

[15] QikProp (2021). Small-Molecule Drug Discovery Suite 2021-1.

[16] Bochevarov A.D., Harder E., Hughes T.F., Greenwood J.R., Braden D.A., Philipp D.M., Rinaldo D., Halls M.D., Zhang J., Friesner R.A. (2013). Jaguar: A high-performance quantum chemistry software program with strengths in life and materials sciences. Int. J. Quantum Chem..

[17] Islam N. (2015). Investigation of comparative shielding of Morin against oxidative damage by radicals: A DFT study. Cogent Chem..

[18] Kremer D., Akkermann R., Küry P., Dutta R. (2019). Current advancements in promoting remyelination in multiple sclerosis. Mult. Scler. J..

[19] Watanabe K., Morinaka Y., Iseki K., Watanabe T., Yuki S., Nishi H. (2003). Structure–activity relationship of 3-methyl-1-phenyl-2-pyrazolin-5-one (edaravone). Redox Rep..

[20] Nevitt T., Ohrvik H., Thiele D.J. (2012). Charting the travels of copper in eukaryotes from yeast to mammals. Biochim. Biophys. Acta.

[21] Singleton V., Orthofer R., Lamuela-Raventos R.M. (1999). Analysis of total phenols and other oxidation substrates and antioxidants by means of Folin-Ciocalteu reagent. Methods Enzymol..

[22] Becke A.D. (1993). Density-functional thermochemistry. III. The role of exact exchange. J. Chem. Phys..

[23] Marenich A.V., Cramer C.J., Truhlar D.G. (2009). Universal solvation model based on solute electron density and on a continuum model of the solvent defined by the bulk dielectric constant and atomic surface tensions. J. Phys. Chem. B.

[24] Holzer W., Plagens B., Lorenz K. (1997). Alkylation of Pyrazolones via the Mitsunobu Reaction. Heterocycles.

[25] Haessner R., Hennig L., Gaca J. (1990). 13C NMR Data for Chlorine- or Nitro-Substituted Azomethine Dyes. Magn. Reson. Chem..

[26] Fan W., Li W., Ma X., Tao X., Li X., Yao Y., Xie X., Zhang Z. (2011). Ru-Catalyzed Asymmetric Hydrogenation of γ-Heteroatom Substituted β-Keto Esters. J. Org. Chem..

[27] Kotha S., Shirbhate M.E. (2012). Diversity-Oriented Approach to Macrocyclic Cyclophane Derivatives via RingClosing Metathesis. Synlett.

