# Supplementary material for: Synthesis and Characterization of Edaravone Analogues as Remyelinating Agents and Putative Mechanistic Probes
Source: Molecules. 2023 Oct 4;28(19):6928. doi: 10.3390/molecules28196928 (PMC10574562; doi:10.3390/molecules28196928)
Supplement: Supplementary file 1 [file molecules-28-06928-s001.zip › molecules-2630085-supplementary.pdf]

Synthesis and Characterization of Edaravone Analogues as Remyelinating Agents and Putative Mechanistic Probes

Eleonora Colombo [1,†,‡], Stefania Olla [2,†], Cristina Minnelli [3], Alessia Formato [4,§], Caterina Veroni [4], Silvia Corbisiero [4,‖], Mattia Pericolo [1], Chiara Siguri [2], Giovanna Mobbili [3], Cristina Agresti [4,*] and Pierfausto Seneci [1,*]

[1] Chemistry Department, University of Milan, Via Golgi 19, 20133 Milan, Italy; ecolombo2@bwh.harvard.edu (E.C.); mattia.pericolo12@gmail.com (M.P.)
[2] Biomedical and Genetic Research Institute (IRGB, National Research Council (CNR)), University Campus, 09042 Monserrato, Italy; stefania.olla@irgb.cnr.it (S.O.); chiara.siguri@irgb.cnr.it (C.S.)
[3] Department of Life and Environmental Sciences, Marche Polytechnic University, Via Brecce Bianche, 60131 Ancona, Italy; c.minnelli@staff.univpm.it (C.M.); g.mobbili@staff.univpm.it (G.M.)
[4] Department of Neuroscience, National Institute of Health (ISS), Viale Regina Elena 299, 00161 Rome, Italy; a.formato92@gmail.com (A.F.); caterina.veroni@iss.it (C.V.); silvia.corbisiero98@gmail.com (S.C.)
[*] Correspondence: cristina.agresti@iss.it (C.A.); pierfausto.seneci@unimi.it (P.S.)
[†] These authors contributed equally to this work.
[‡] Current address: Harvard Medical School and Brigham and Women's Hospital, Boston, MA 02115, USA
[§] Current address: Institute of Biochemistry and Cell Biology, IBBC-CNR, Campus Adriano Buzzati Traverso, Via Ercole Ramarini 32, Monterotondo Scalo, 00015 Rome, Italy
[‖] Current address: Institute for Treatment and Research (IRCCS) Santa Lucia Foundation, Neuroimmunology Unit, 00143 Rome, Italy

SUPPLEMENTARY INFORMATION

Chapter 1: Medicinal Chemistry

1.1 General information

Commercially available reagents (Sigma Aldrich) were used without further purification. Reactions requiring anhydrous conditions were performed under nitrogen atmosphere. THF, CH_2Cl_2 and methanol were dried using molecular sieves (3 Å beads, 8-12 mesh). Dry THF, CH_2Cl_2, methanol, DMF, DME and DIPEA were purchased from Sigma Aldrich.

Thin layer chromatography

Reactions and chromatography purifications were monitored by TLC using silica gel pre-coated glass plates (60 F254, Sigma-Aldrich, 0.25 mm thickness). TLC spots were visualized with UV lamps (254 υn), or with staining reagents such as *Potassium permanganate* (5 g $KMnO_4$, 30 g K_2CO_3, 7.5 mL NaOH 5%, 400 mL H_2O).

Chromatographic purification

Purification of intermediates and final products was mostly carried out by flash chromatography using as stationary phase high purity grade silicagel (Merck Grade, pore size 60 Å, 230-400 mesh particle size, Sigma Aldrich). Alternatively, purification was performed by a Biotage® system, both in normal and reverse phase.

Biotage® KP-C18-HS cartridges (6-12 g) were used for reverse phase chromatography. The elution method was as follows: flow: 6-15 mL/min; equilibration time of 2 min; monitoring wavelength: 210 nm, 254 nm; phases: H_2O (HiPerSolv Chromanorm VWR water for HPLC-MS) (A), acetonitrile (HiPerSolv Chromanorm VWR acetonitrile SuperGradient) (B); elution gradient: 0% B for 2 min, then 0% B to 100% B in 20 min, then 100% B for 2 min.

Biotage® Sfär Silica D cartridges (10/25 g) were used for direct phase chromatography. The elution method was as follows: flow 40-80 mL/min; equilibration time of 2 min; monitoring wavelength: 210 nm, 254 nm; phases: n-hexane (HiPerSolv Chromanorm VWR n-hexane for HPLC-MS) (A), ethyl acetate (HiPerSolv Chromanorm VWR ethyl acetate SuperGradient) (B); or dichloromethane (HiPerSolv Chromanorm VWR dichloromethane for HPLC-MS) (A), methanol (HiPerSolv Chromanorm VWR methanol SuperGradient) (B).

Liquid chromatography and mass spectrometry

Ultra-high Performance Liquid Chromatography/Mass Spectrometry (UPLC/MS) was performed with an Acquity™ UPLC/MS System equipped with a TUV Detector, a single quadrupole SQD mass spectrometer and ACQUITY UPLC BEH SHIELD RP_{18} columns (2.1x100mm, id = 1.7 μm). The elution method was as follows: flow: 0.5 mL/min, with equilibration time of 2 min; monitoring wavelength: 220 nm; column temperature 40°C, sample temperature 25°C; phases: water (HiPerSolv Chromanorm VWR water for HPLC-MS) + 0,05% trifluoroacetic acid (spectroscopic grade) (A), acetonitrile (HiPerSolv Chromanorm VWR acetonitrile SuperGradient) + 0,05% trifluoroacetic acid (spectroscopic grade) (B): *elution gradient 1*: 0% B to 40% B in 5 min, then 100% B for 1 min; *elution gradient 2*: 5% B to 100% B in 5 min, then 100% B for 1 min; *elution gradient 3*: 40% B to 100% B in 5 min, then 100% B for 1 min;

MS-SCAN: 100-1000 uma, SCAN-time: 0.2 sec. ESI^+ was used as an ionization technique for MS.

NMR spectroscopy

Intermediates and final products were structurally characterized by ^1H NMR and ^{13}C NMR spectroscopy at 300/400 MHz, using a Bruker AC 300/400 spectrometer. Chemical shifts were expressed in ppm relative to internal Me_4Si as standard.

^1H-NMR spectra are described reporting for each signal: chemical shift; multiplicity; integration; attribution (using bi-dimensional experiments COSY and HSQC); coupling constants. ^{13}C-NMR spectra are described reporting for each signal their chemical shift.

1.2 Synthesis and analytical characterization

Methyl 4-(3-methyl-5-oxo-4,5-dihydro-1H-pyrazol-1-yl)benzoate 2c

The synthesis of edaravone ester **2c** is described in the main text.

Analytical characterization

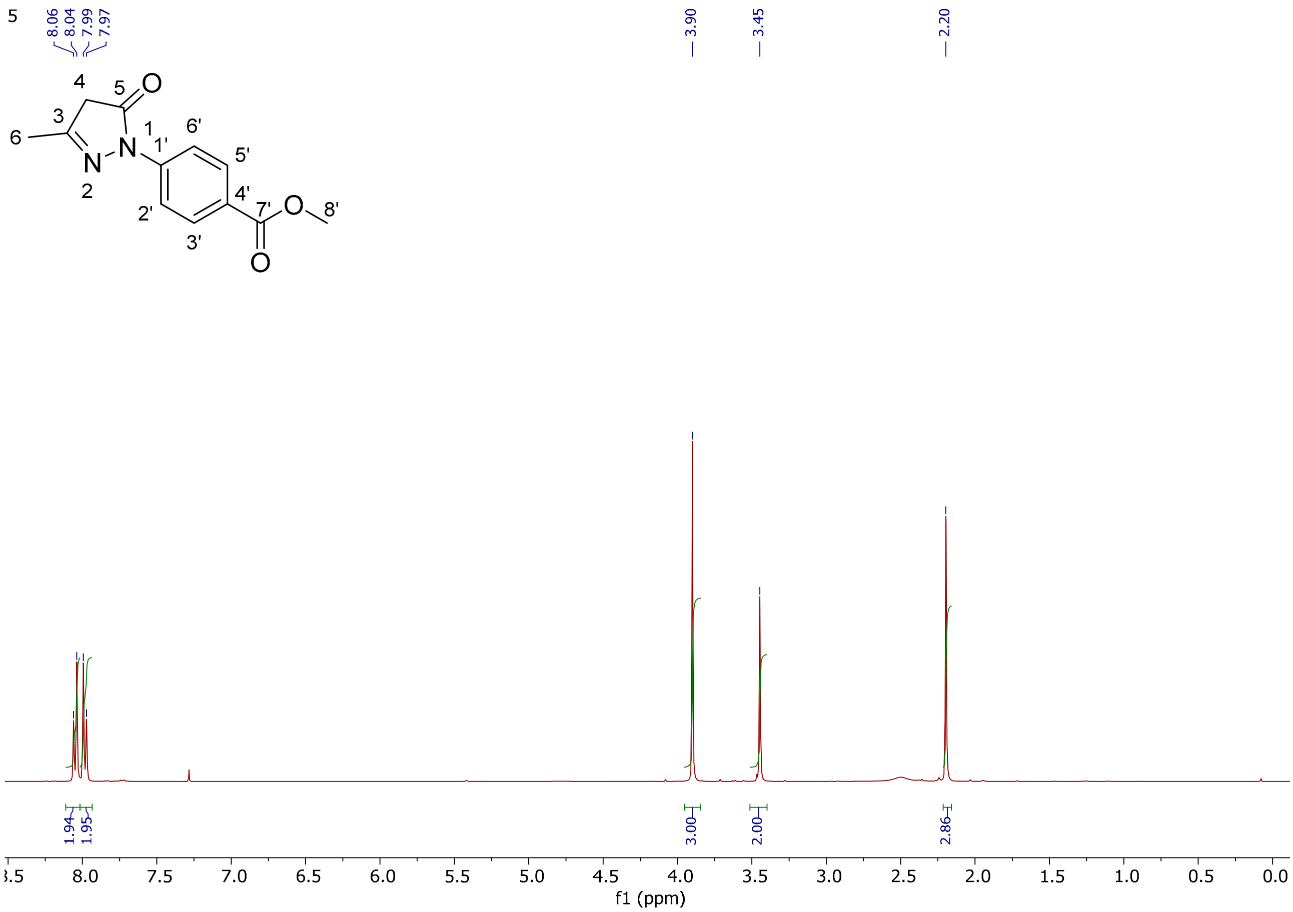

^{1}H-NMR (400 MHz, $CDCl_3$): δ (ppm) 8.05 (d, *J* = 9.0 Hz, 2H, **H3'-H5'**), 7.98 (d, *J* = 9.0 Hz, 2H, **H2'-6'**), 3.90 (s, 3H, **H8'**), 3.45 (s, 2H, **H4**), 2.20 (s, 3H, **H6**).

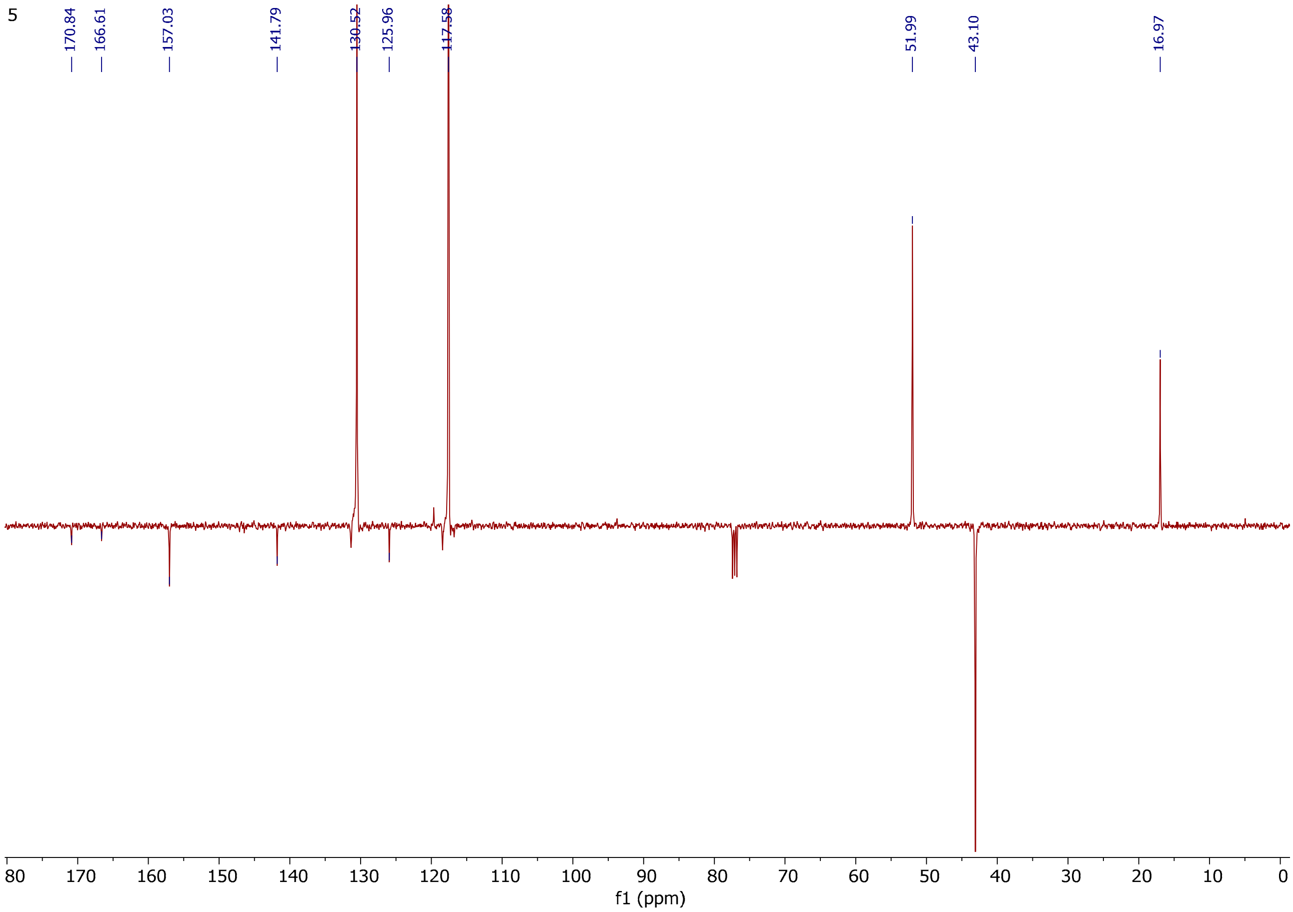

^{13}C-NMR (101 MHz, $CDCl_3$): δ (ppm) 170.8, 166.6, 157.0, 141.8, 130.5 (2C), 126.0, 117.6 (2C), 52.0, 43.1, 17.0.

MS (ESI), m/z: calcd for $C_{12}H_{12}N_2O_3$ 232.08, found 233.33 ($M+H^+$).

Synthesis of *N*-(2-azidoethyl)-4-(3-methyl-5-oxo-4H-pyrazol-1-yl)benzamide 2d

EDC (66.2 mg, 0.344 mmol), DMAP (84.7 mg, 1.03 mmol) and 2-azidoethanamine (56.4 mg, 0.458 mmol) were added to a stirred solution of carboylate **2b** (50.0 mg, 0.229 mmol) in dry CH_2Cl_2 (5 mL) under nitrogen atmosphere. The mixture was left stirring at rt overnight. Reaction monitoring (TLC, eluent mixture: 1:9 n-hex/AcOEt + 1% formic acid) confirmed the disappearance of **2**. The reaction mixture was washed with saturated aqueous NH_4Cl (5 mL) and then with saturated aqueous $NaHCO_3$ (5 mL). The aqueous phase was extracted with CH_2Cl_2 (3 x 5 mL). The collected organic extracts were dried with Na_2SO_4 and evaporated under reduced pressure. The resulting crude was purified by flash chromatography (silicagel, eluent mixture: 2:8 n-hex/AcOEt) to obtain pure title compound **2d** (53.2 mg, 0.185 mmol, 80% yield) as a yellow solid.

Analytical characterization

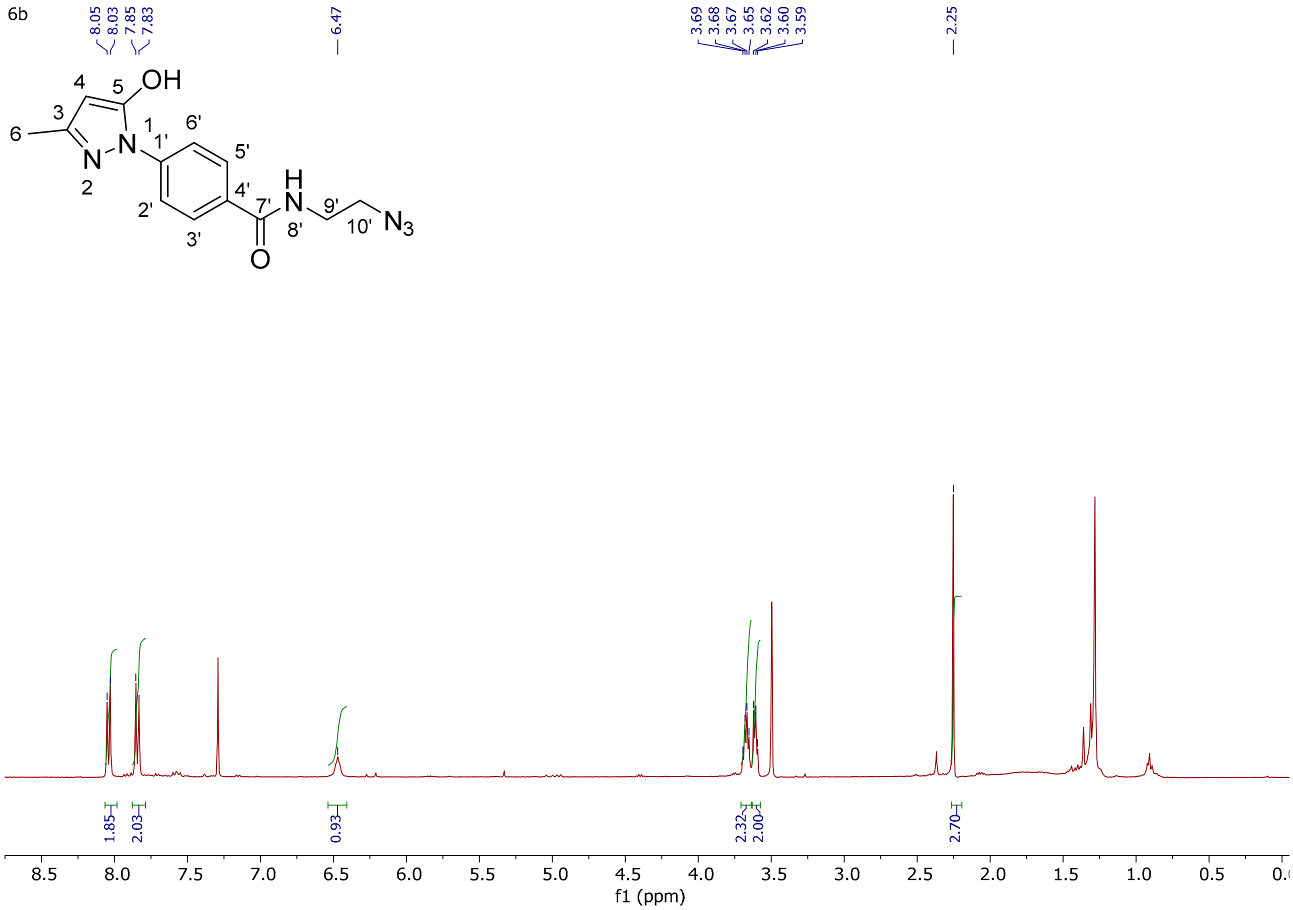

¹H-NMR (400 MHz, $CDCl_3$): δ (ppm) 8.04 (d, *J* = 8.7 Hz, 2H, **H3'-H5'**), 7.84 (d, *J* = 8.7 Hz, 2H, **H2'-H6'**), 6.47 (bs, 1H, **H4**), 3.69 – 3.65 (m, 2H, **H9'**), 3.62 – 3.59 (m, 2H, **H10'**), 2.25 (s, 3H, **H6**).

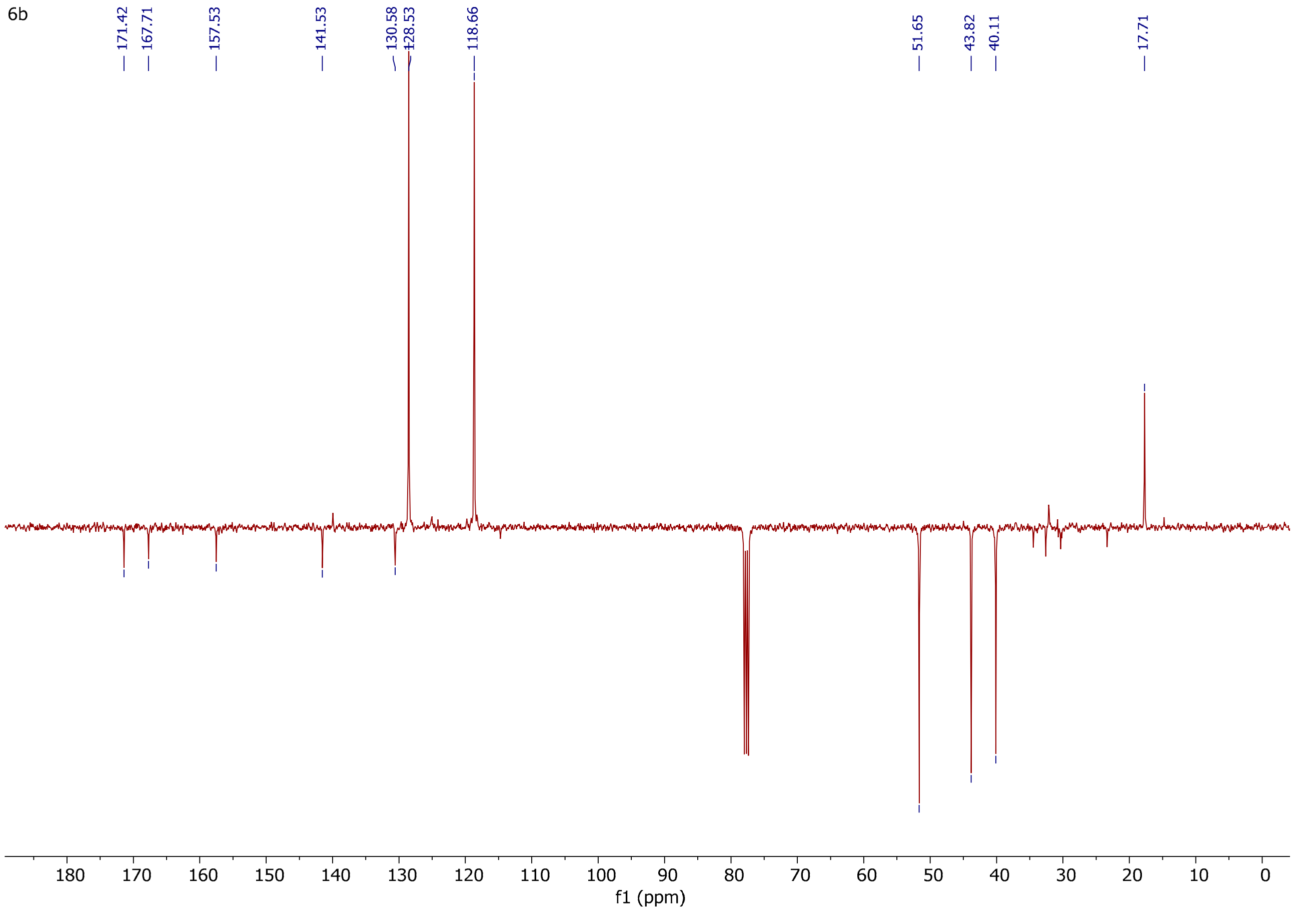

^{13}C-NMR (101 MHz, $CDCl_3$): δ (ppm) 171.4, 167.7, 157.5, 141.5, 130.6, 128.5 (2C), 118.7 (2C), 51.6, 43.8, 40.1, 17.7.

MS (ESI), m/z: calcd for $C_{13}H_{14}N_6O_2$ 268.12, found 269.28 ($M+H^+$).

Synthesis of *N*-(6-azidohexyl)-4-(3-methyl-5-oxo-4H-pyrazol-1-yl)benzamide 2e

EDC (263 mg, 1.37 mmol), DMAP (336 mg, 2.75 mmol) and 6-azidohexylamine (260 mg, 1.83 mmol) were added to a stirred solution of carboxylate **2b** (200 mg, 0.916 mmol) in dry CH_2Cl_2 (20 mL) under nitrogen atmosphere. The mixture was left stirring at rt overnight. Reaction monitoring (TLC, eluent mixture: 98:2 CH_2Cl_2/MeOH) confirmed the disappearance of **2**. The reaction mixture was quenched with the addition of 0.1 M HCl (5 mL) and left stirring for 1 h. The aqueous phase was then washed with 1M HCl (10 mL) and extracted with CH_2Cl_2 (3 x 10 mL). The collected organic extracts were dried with Na_2SO_4 and evaporated under reduced pressure. The resulting crude was purified by reverse phase chromatography (Biotage®, eluent mixture water/ACN from 10 to 100%) to obtain pure title compound **2e** (162 mg, 0.474 mmol, 52% yield) as a yellow solid.

Analytical characterization

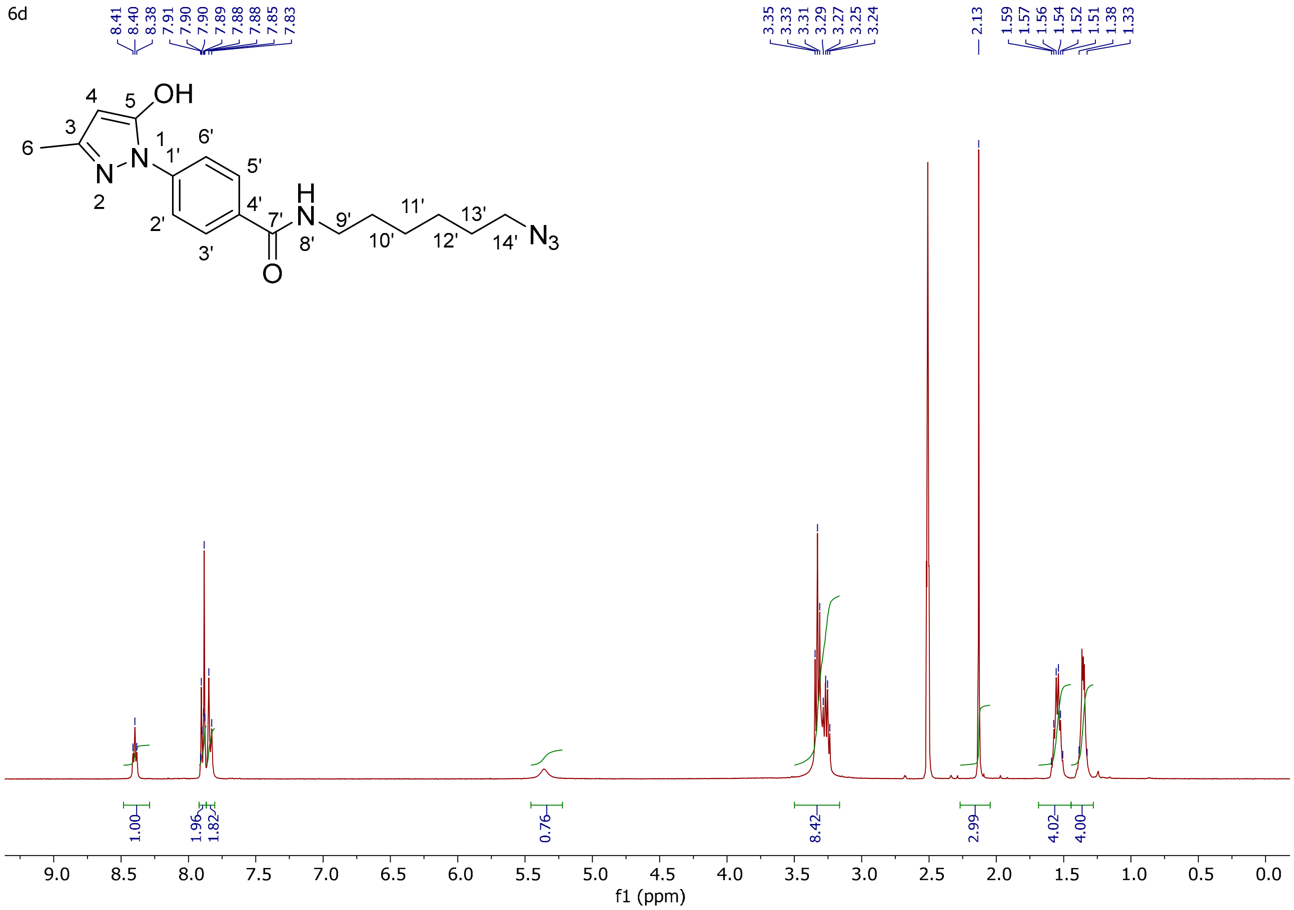

^{1}H-NMR (400 MHz, DMSO-d$_6$): δ (ppm) 8.40 (t, *J* = 5.5 Hz, 1H, **H8'**), 7.92 – 7.87 (m, , *J* = 8.7 Hz, 2H, **H3'-H5'**), 7.84 (d, *J* = 8.7 Hz, 2H, **H2'-H6'**), 5.36 (s, 1H, **H4**), 3.40 – 3.18 (m, 4H, **H9'-H14'**), 2.13 (s, 3H, **H6**), 1.60 - 1.49 (m, 4H, **H10'-H13'**), 1.49 – 1.29 (m, 4H, **H11'-H12'**).

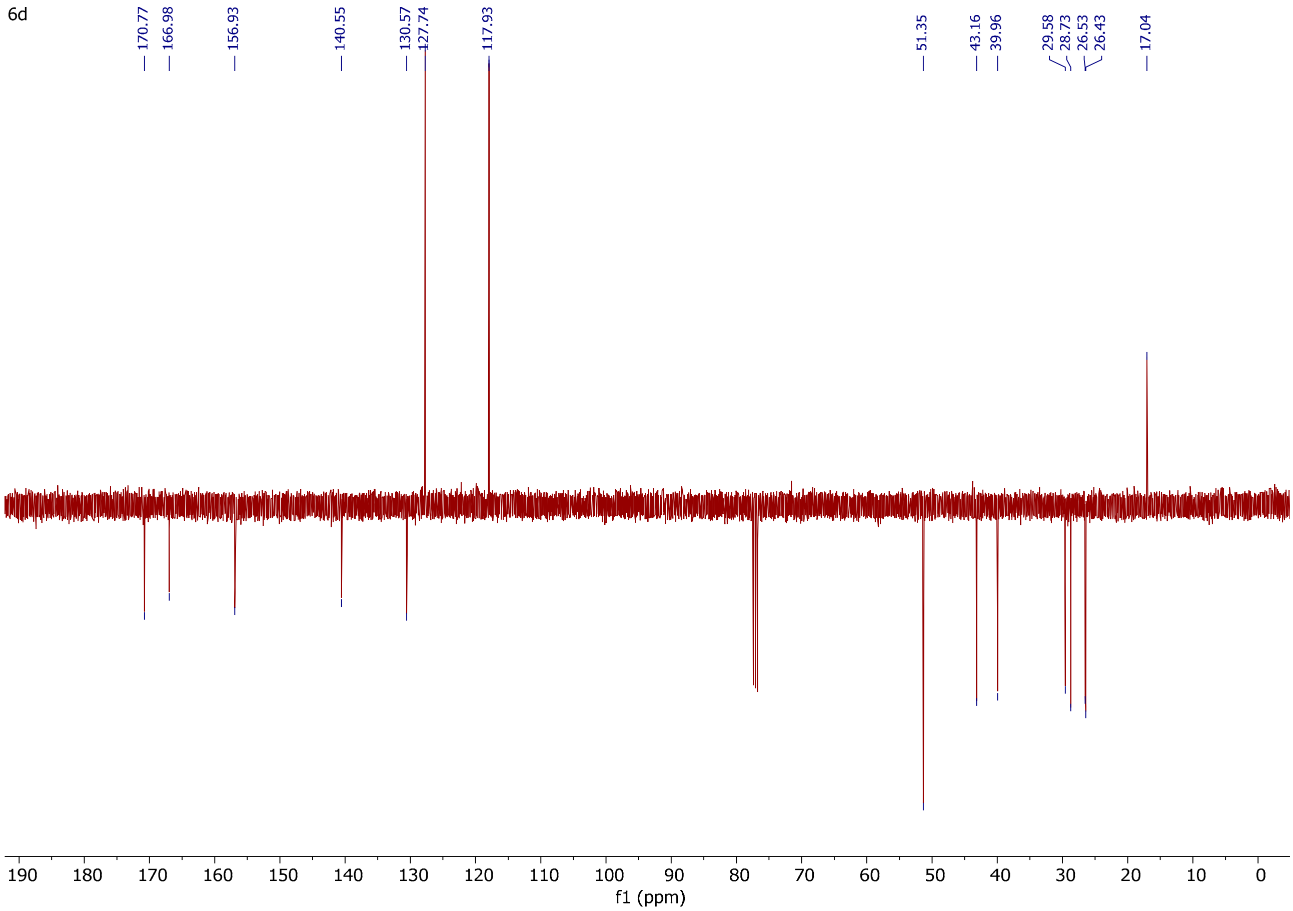

^{13}C-NMR (101 MHz, $CDCl_3$): δ (ppm) 170.8, 167.0, 156.9, 140.6, 130.6, 127.7 (2C), 117.9 (2C), 51.4, 43.2, 40.0, 29.6, 28.7, 26.5, 26.4, 17.0.

MS (ESI), m/z: calcd for $C_{17}H_{22}N_6O_2$ 342.18, found 343.25 ($M+H^+$).

Synthesis of 4-(3-methyl-5-oxo-4,5-dihydro-1H-pyrazol-1-yl)-N-(4-(3-(trifluoromethyl)-3H-diazirin-3-yl)benzyl)benzamide 2f

EDC·HCl (130 mg, 0.687 mmol), DMAP (84 mg, 0.687 mmol) and (4-(3-(trifluoromethyl)-3H-diazirin-3-yl)phenyl)methanamine (230 mg, 0.916 mmol) were added under nitrogen atmosphere to a stirred solution of carboxylate **2b** (100 mg, 0.458 mmol) in dry CH_2Cl_2 (10 mL). The reaction mixture was stirred at rt for 12 h. Reaction monitoring (TLC, eluent mixture: 95:5 CH_2Cl_2/MeOH) confirmed the disappearance of **2**. The reaction mixture was then washed with saturated aqueous NH_4Cl (10 mL) and with saturated aqueous $NaHCO_3$ (10 mL). The collected aqueous phase was extracted with CH_2Cl_2 (3 x 12 mL). The organic extracts were dried with Na_2SO_4 and evaporated under reduced pressure. The crude was purified by reverse phase chromatography (Biotage®, eluent mixture H_2O/ACN). Pure title compound **2f** was obtained as a white solid (29,2 mg, 0.069 mmol, 15% yield).

Analytical characterization

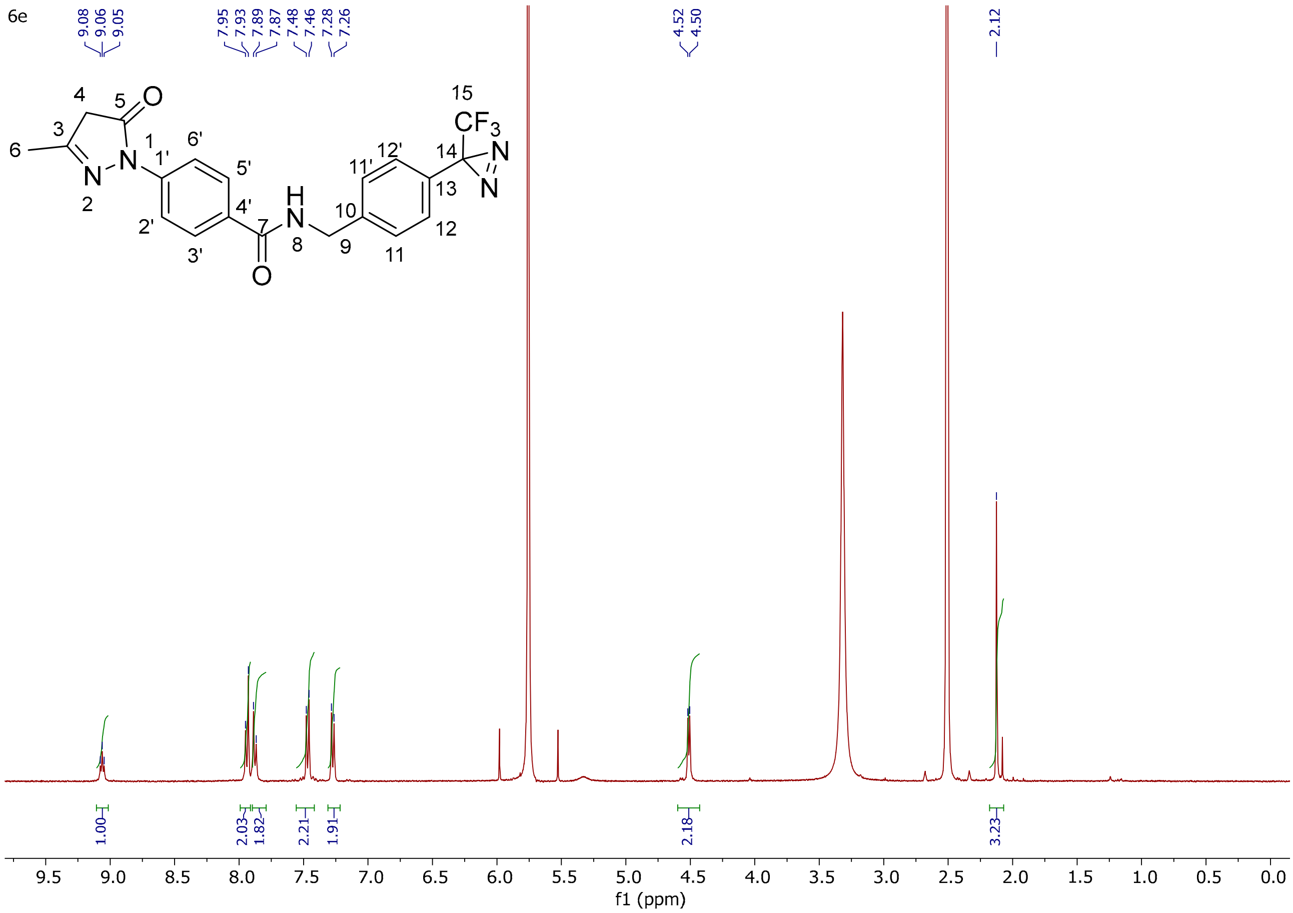

^{1}H-NMR (400 MHz, $CDCl_3$): δ (ppm) 9.06 (t, 1H, *J* = 6.0 Hz, **H8**), 7.94 (d, 2H, *J* = 8.8 Hz, **H11-H11'**), 7.88 (d, 2H, *J* = 8.6 Hz, **H12-H12'**), 7.47 (d, 2H, *J* = 8.4 Hz, **H3'-H5'**), 7.27 (d, 2H, *J* = 8.0 Hz, **H2'-H6'**), 4.51 (d, 2H, *J* = 5.7 Hz, **H9**), 2.12 (s, 3H, **H6**).

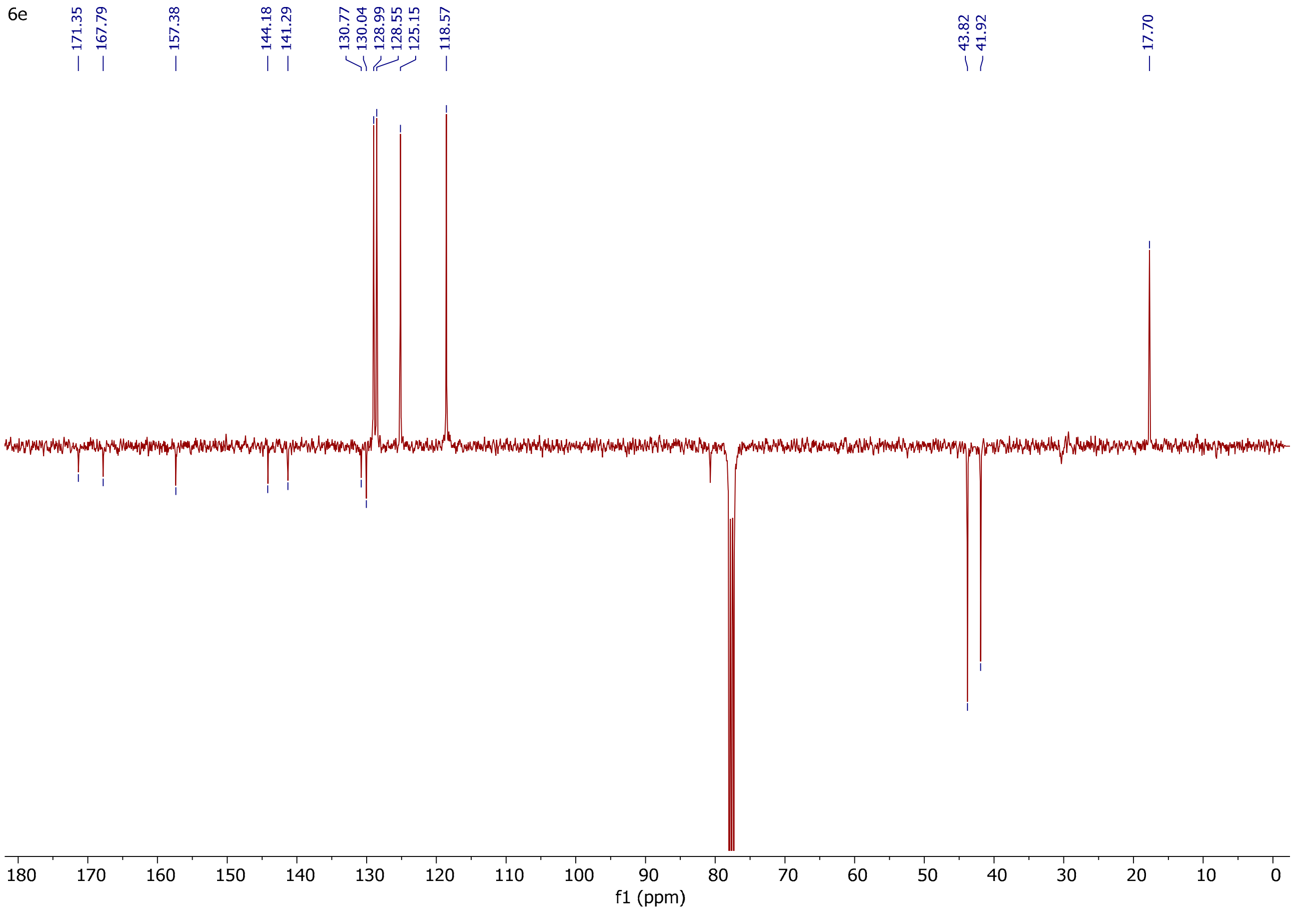

^{13}C-NMR (101 MHz, $CDCl_3$): δ (ppm) 171.4, 167.8, 157.4, 144.2, 141.3, 130.8, 130.0, 129.0 (2C), 128.6 (2C), 125.2 (2C), 118.6 (2C), 43.8, 41.9, 17.7.

MS (ESI), m/z: calcd for $C_{20}H_{16}F_3N_5O_2$ 415.13, found 416.40 ($M+H^+$).

Synthesis of tert-butyl 2-(4-(methoxycarbonyl)phenyl)-5-methyl-3-oxo-2,3-dihydro-1H-pyrazole-1-carboxylate 4a

The synthesis of Boc edaravone ester **4a** is described in the main text.

Analytical characterization

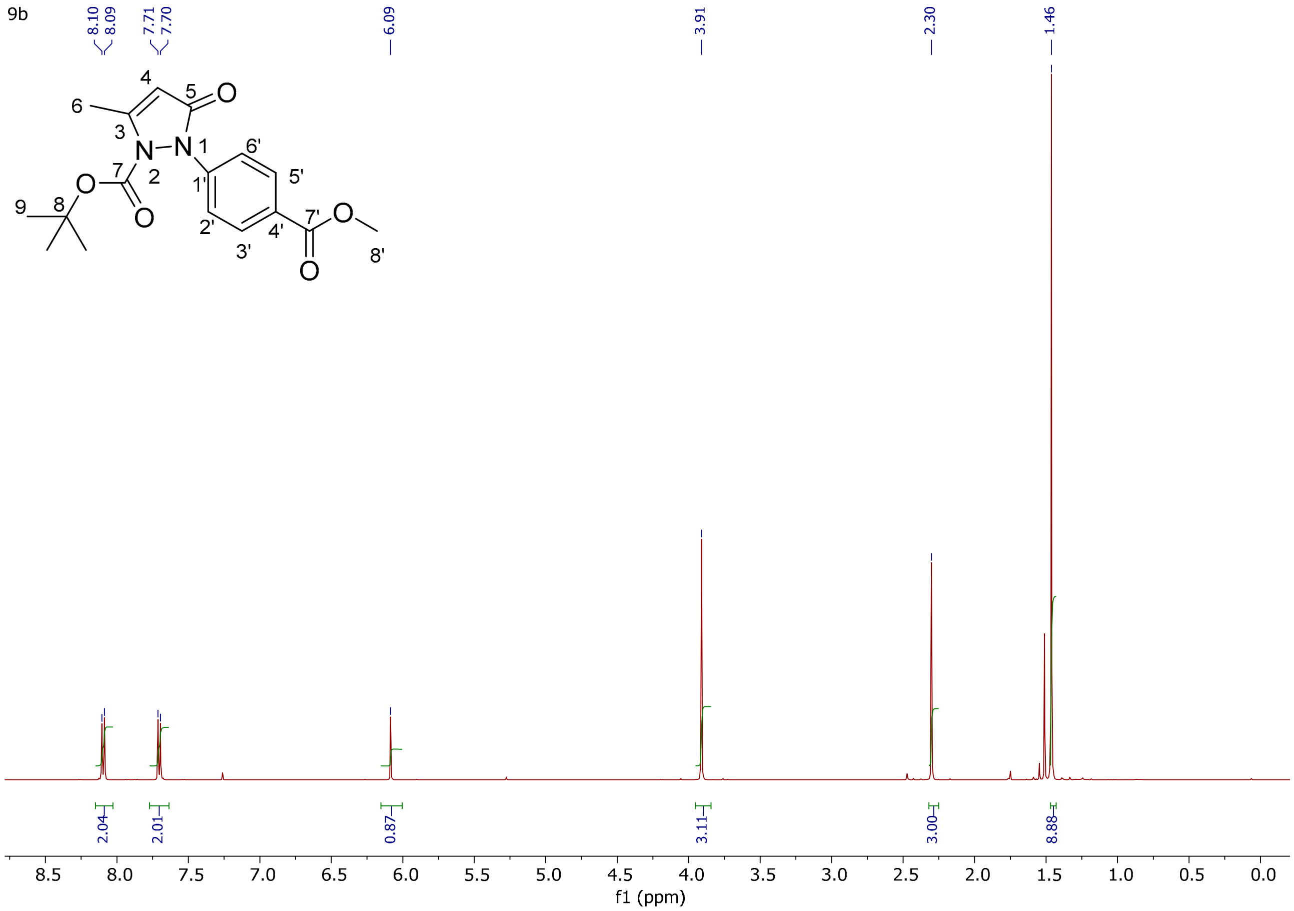

^{1}H-NMR (500 MHz, $CDCl_3$): δ(ppm) 8.10 (d, 2H, *J* = 8.8 Hz, **H3'-H5'**), 7.70 (d, 2H, *J* = 8.8 Hz, **H2'-H6'**), 6.09 (s, 1H, **H4**), 3.91 (s, 3H, **H11**), 2.30 (s, 3H, **H5**), 1.46 (s, 9H, **H9**).

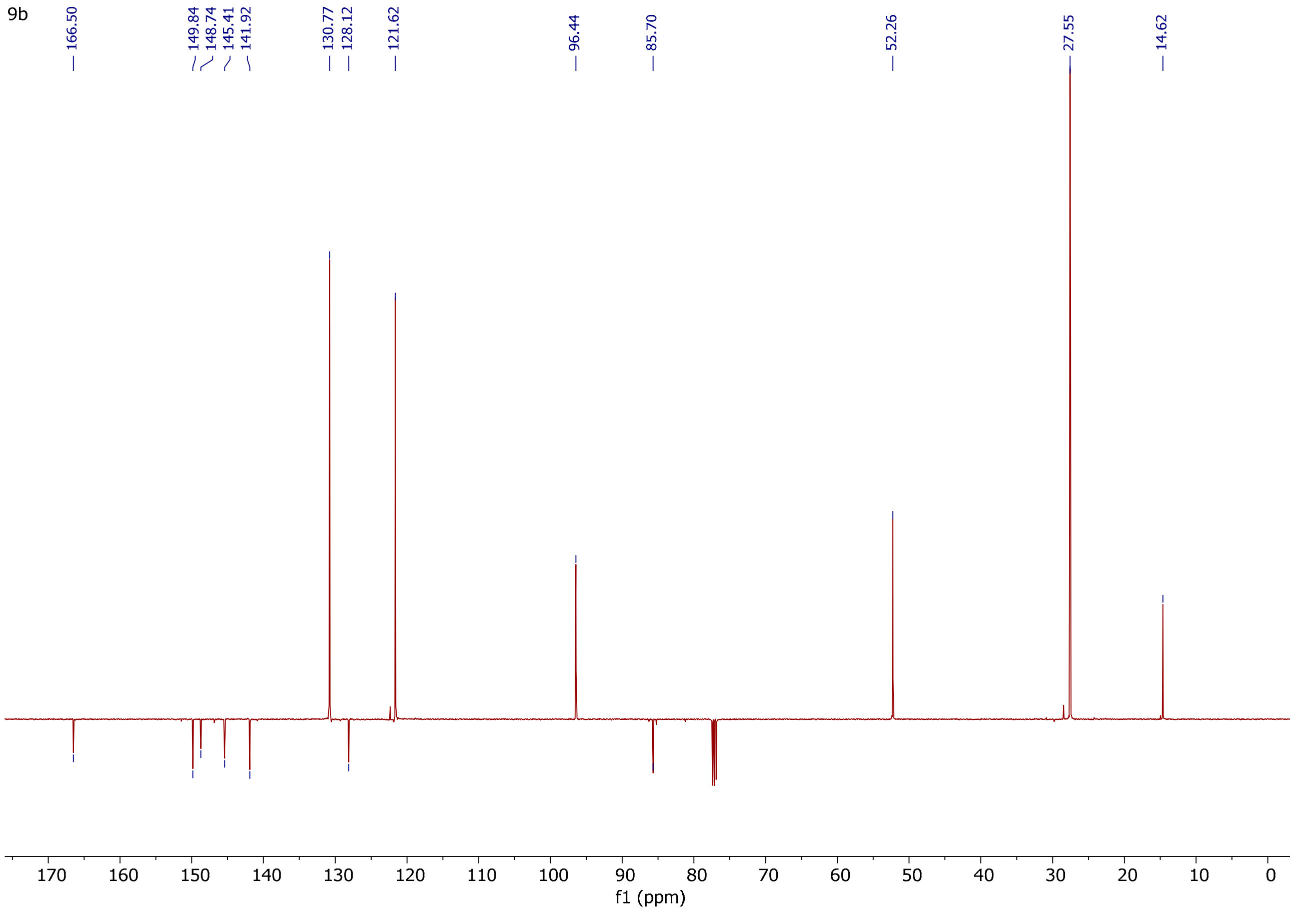

^{13}C-NMR (126 MHz, $CDCl_3$): δ (ppm) 166.5, 149.8, 148.7, 145.4, 141.9, 130.8 (2C), 128.1, 121.6 (2C), 96.4, 85.7, 52.3, 27.6 (3C), 14.6.

MS (ESI), m/z: calcd for $C_{17}H_{20}N_2O_5$ MW 332.14, found 333.26 ($M+H^+$).

Synthesis of tert-butyl 2-(4-(hydroxymethyl)phenyl)-5-methyl-3-oxo-2,3-dihydro-1H-pyrazole-1-carboxylate 4b

1M DIBAL-H in THF (2.3 mL, 2.37 mmol) was added under nitrogen atmosphere to a stirred solution of carbamate ester **4a** (315 mg, 0.948 mmol) in dry CH_2Cl_2 (9.4 mL) at -20°C. Then the reaction mixture was stirred at rt for 10 h, monitoring the reaction with TLC (eluent mixture 8:2 n-hex/AcOEt) until disappearance of **4a**. The reaction mixture was then quenched with MeOH (142 µL) and saturated aq. Na_2SO_4 (619 µL) at -20°C for 30 min. Solid Na_2SO_4 (6.19 g) was added, then the resulting suspension was filtered on celite. The plug was washed with CH_2Cl_2 (10 mL), then the collected organic phase was evaporated under reduced pressure to a crude which was purified by flash chromatography (silicagel, eluent mixture 8:2 n-hex/AcOEt). Pure title compound **4b** was obtained as a white solid (154 mg, 0.507 mmol, 54% yield).

Analytical characterization

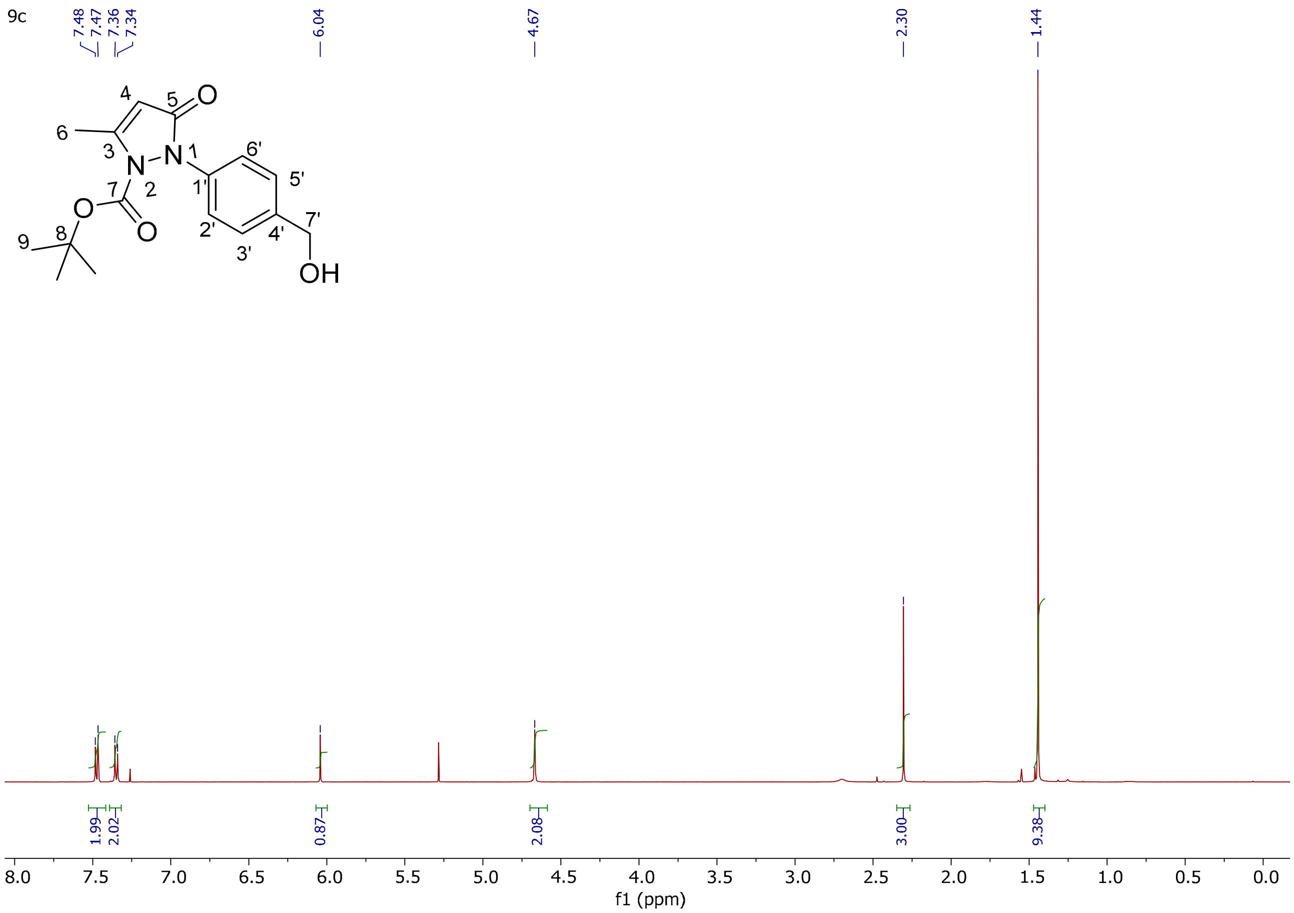

^{1}H-NMR (500 MHz, $CDCl_3$): δ (ppm) 7.47 (d, 2H, *J* = 8.6 Hz, **H3'-H5'**), 7.35 (d, 2H, *J* = 8.7 Hz, **H2'-H6'**), 6.04 (s, 1H, **H4**), 4.67 (s, 2H, **H10**), 2.70 (s, 1H, **OH)**, 2.30 (s, 3H, **H5**), 1.44 (s, 9H, **H9**).

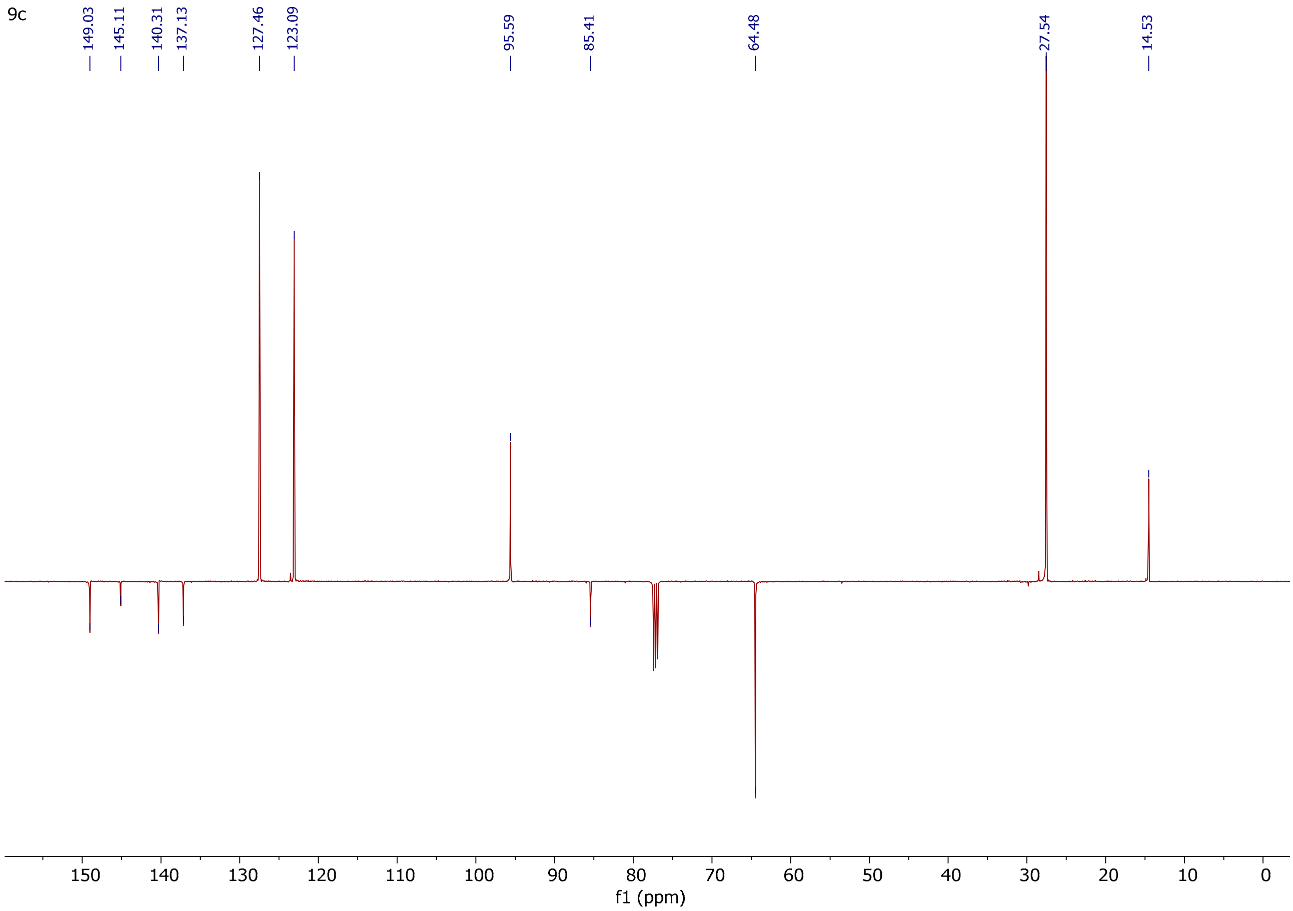

^{13}C-NMR (126 MHz, $CDCl_3$): δ(ppm) 149.0, 145.1, 140.3, 137.1 (2C), 127.5 (2C), 123.1 (2C), 95.6, 85.4, 64.5, 27.5, 14.5.

MS (ESI), m/z: calcd for $C_{17}H_{20}N_2O_5$ MW 304.14, found 305.25 ($M+H^+$).

Synthesis of 2-(4-(hydroxymethyl)phenyl)-5-methyl-2,4-dihydro-3H-pyrazol-3-one 2g

TFA (0.251 mL, 2.95 mmol) was added under nitrogen atmosphere to a stirred solution of carbamate alcohol **4b** (50.0 mg, 0.164 mmol) in dry CH_2Cl_2 (1.6 mL) at -30°C. The reaction mixture was stirred at -30°C for 1 h, then the solvent and TFA were evaporated under reduced pressure to a crude which was purified by flash chromatography (silicagel, eluent mixture 7:3 n-hex/AcOEt). Pure title compound **2g** was obtained as a white solid (20.4 mg, 0.0984 mmol, 60% yield).

Analytical characterization

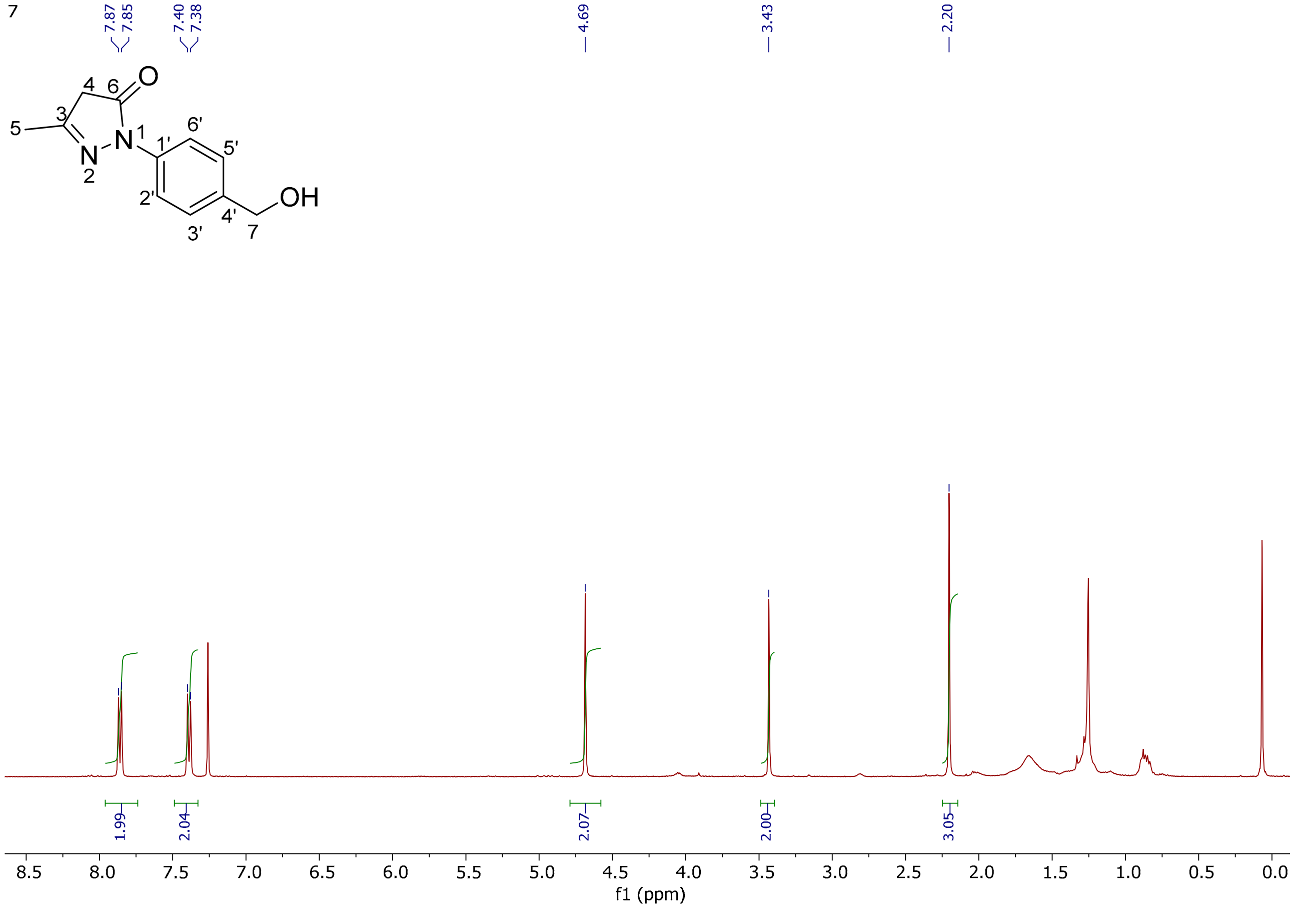

^{1}H-NMR (400 MHz, $CDCl_3$): δ(ppm) 7.86 (d, 2H, *J* = 8.7 Hz, **H3'-H5'**), 7.39 (d, 2H, *J* = 8.9 Hz, **H2'-H6'**), 4.69 (s, 2H, **H7**), 3.43 (s, 2H, **H4**), 2.20 (s, 3H, **H5**).

7

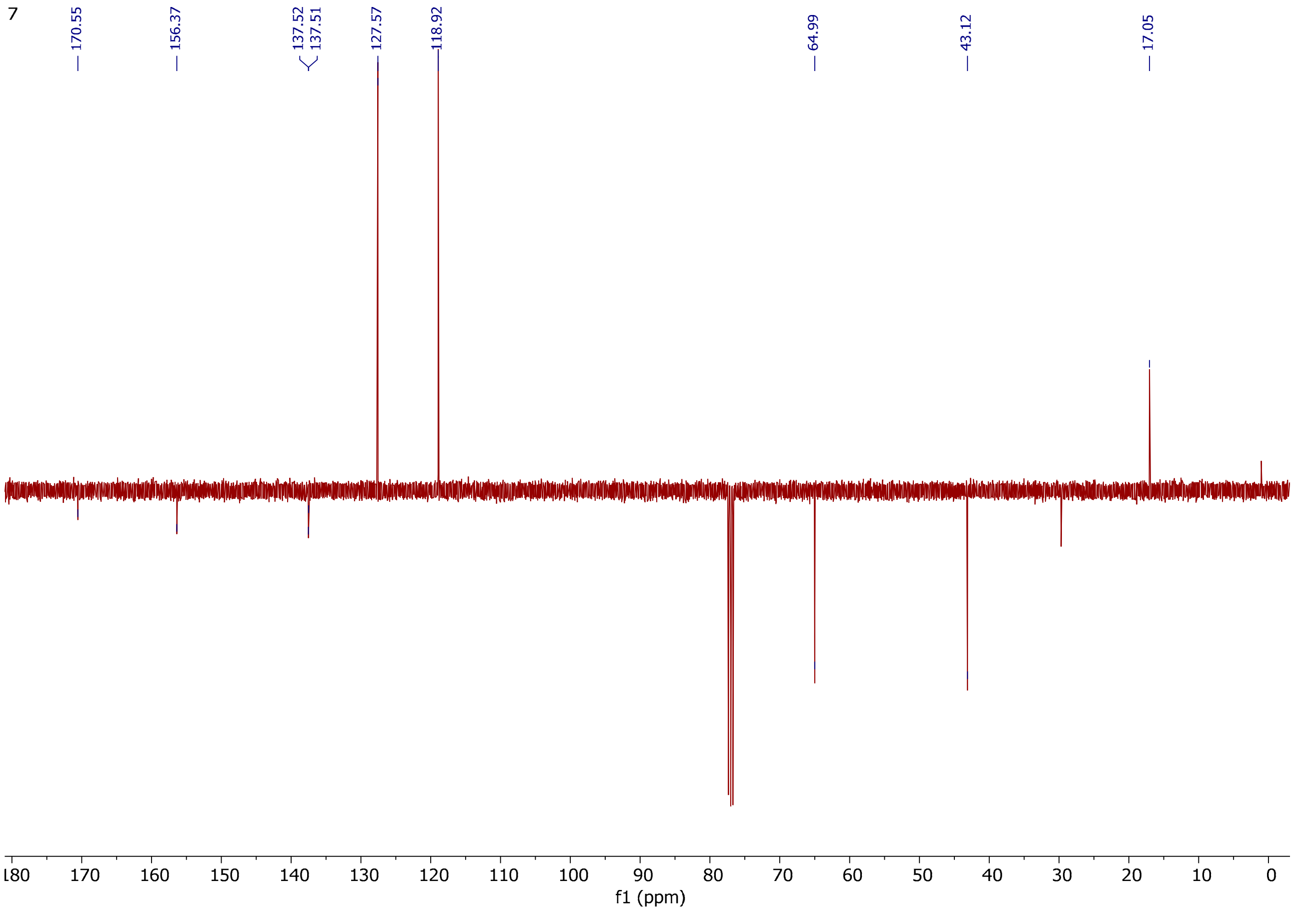

^{13}C-NMR (101 MHz, $CDCl_3$): δ(ppm) 170.6, 156.4, 137.5, 137.5, 127.6 (2C), 118.9 (2C), 65.0, 43.1, 17.0.

MS (ESI), m/z: calcd for $C_{11}H_{12}N_2O_2$ MW 204.09, found 205.15 ($M+H^+$).

Synthesis of tert-butyl 2-(4-(azidomethyl)phenyl)-5-methyl-3-oxo-2,3-dihydro-1H-pyrazole-1-carboxylate 4c

TEA (0.064 mL, 0.456 mmol) was added under nitrogen atmosphere to a stirred solution of carbamate alcohol **4b** (70.0 mg, 0.228 mmol) in dry CH_2Cl_2 (1.15 mL) at -40°C; then, methanesulfonyl chloride (25 [illegible]L, 0.319 mmol) was added, and stirring was continued for 45 min. The reaction was monitored with TLC (eluent mixture 7:3 n-hex/AcOEt), until complete disappearance of **4b**. Then, after warming to rt, the solvent was evaporated with a nitrogen flush, the resulting solid was dissolved in dry DMSO (2 mL), and sodium azide (29.6 mg, 0.456 mmol) was added to the stirred solution. The reaction mixture was stirred at rt for 18 h and was monitored with TLC (eluent mixture 8:2 n-hex/AcOEt) until disappearance of the mesylate. Then, the reaction mixture was diluted with water (5 mL), and the aqueous phase was extracted with AcOEt (3 x 10 mL). The collected organic extracts were dried with Na_2SO_4 and evaporated under reduced pressure to a crude which was purified by flash chromatography (silicagel, eluent mixture 7:3 n-hex/AcOEt). Pure title compound **4c** was obtained as a yellow solid (34.1 mg, 0.103 mmol, 45% yield).

Analytical characterization

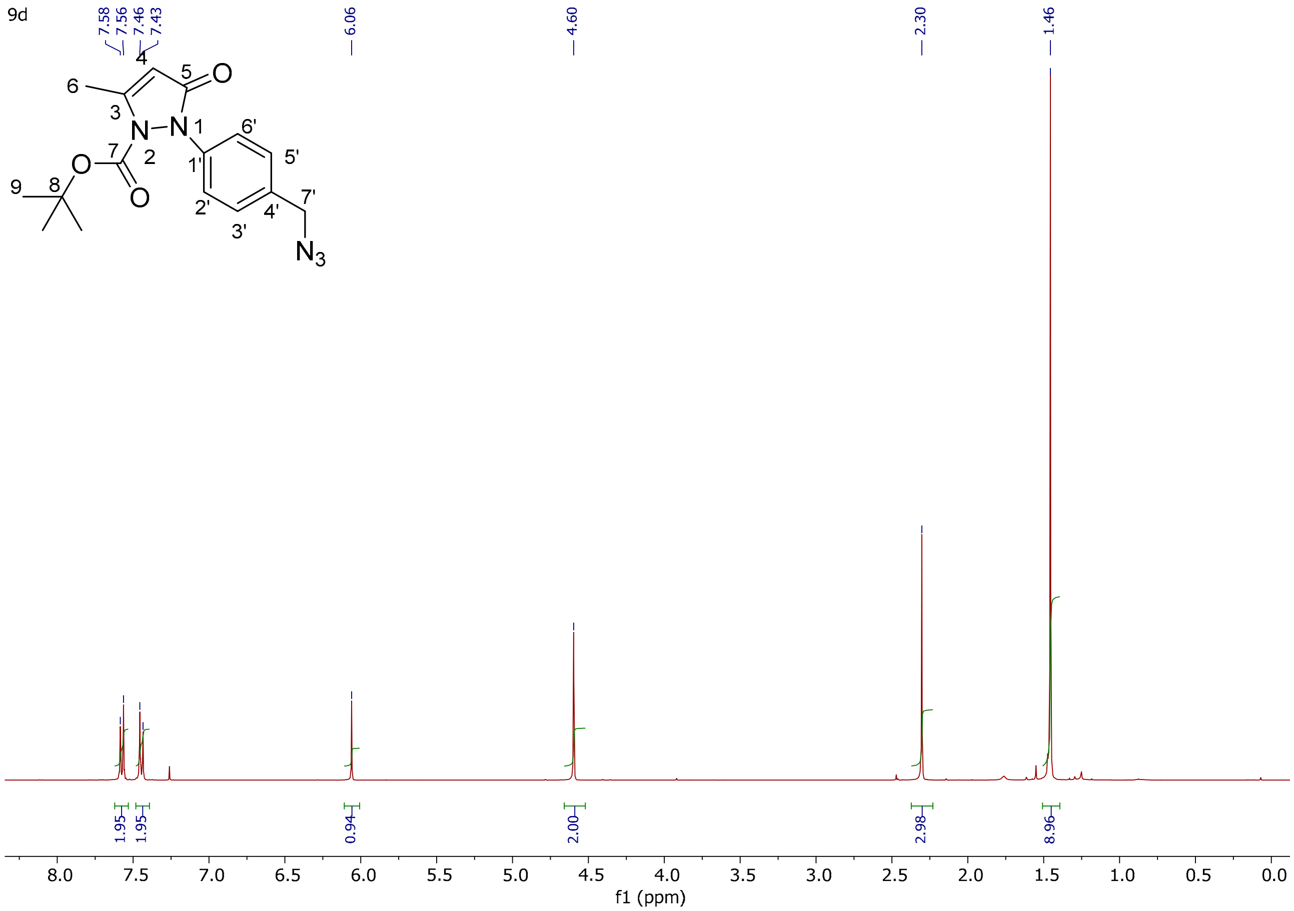

^{1}**H-NMR** (400 MHz, $CDCl_3$): δ (ppm) 7.60 (d, 2H, *J* = 8.6 Hz, **H3'-H5'**), 7.47 (d, 2H, *J* = 8.6 Hz, **H2'-H6'**), 6.08 (s, 1H, **H4**), 4.62 (s, 2H, **H10**), 2.33 (s, 3H, **H5**), 1.48 (s, 9H, **H9**).

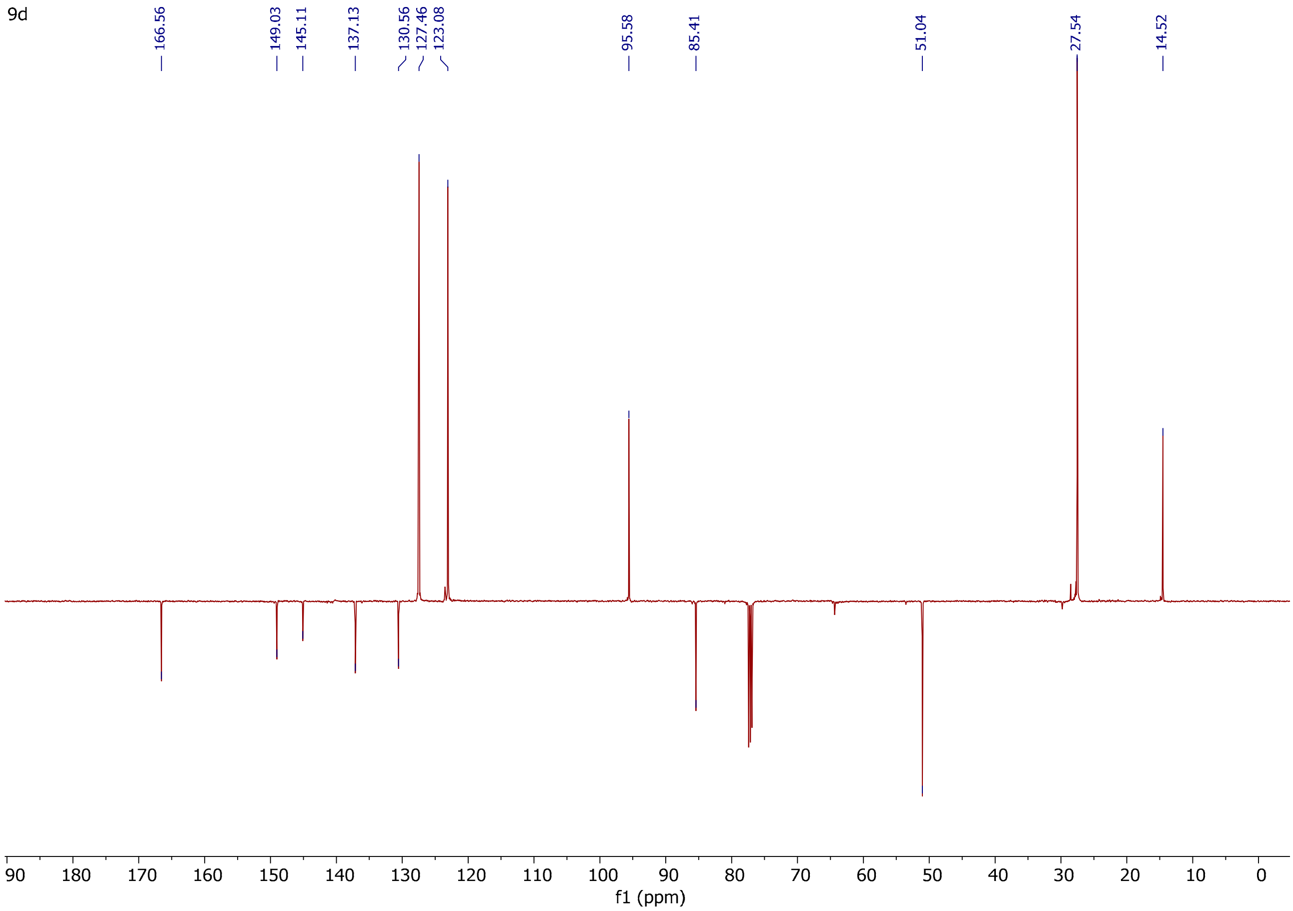

^{13}C-NMR (101 MHz, $CDCl_3$): δ(ppm) 166.6, 149.0, 145.1, 137.1, 130.6, 127.5 (2C), 123.1 (2C), 95.6, 85.4, 51.0, 27.5 (3C), 14.5.

MS (ESI), m/z: calcd for $C_{16}H_{19}N_5O_3$ MW 329.15, found 330.16 ($M+H^+$).

Synthesis of 2-(4-(azidomethyl)phenyl)-5-methyl-2,4-dihydro-3H-pyrazol-3-one 2h

TFA (0.324 mL, 4.24 mmol) was added under nitrogen atmosphere to a stirred solution of carbamate alcohol **4c** (70.0 mg, 0.210 mmol) in dry CH_2Cl_2 (2.1 mL) at -30°C. The reaction was gently warmed to 0°C under stirring in 3 h. Then, the solvent and TFA were evaporated under reduced pressure to a crude which was purified with flash chromatography (silicagel, eluent mixture 7:3 n-hex/AcOEt). Pure title compound **2h** was obtained as a yellow solid (43.3 mg, 0.189 mmol, 90% yield).

Analytical characterization

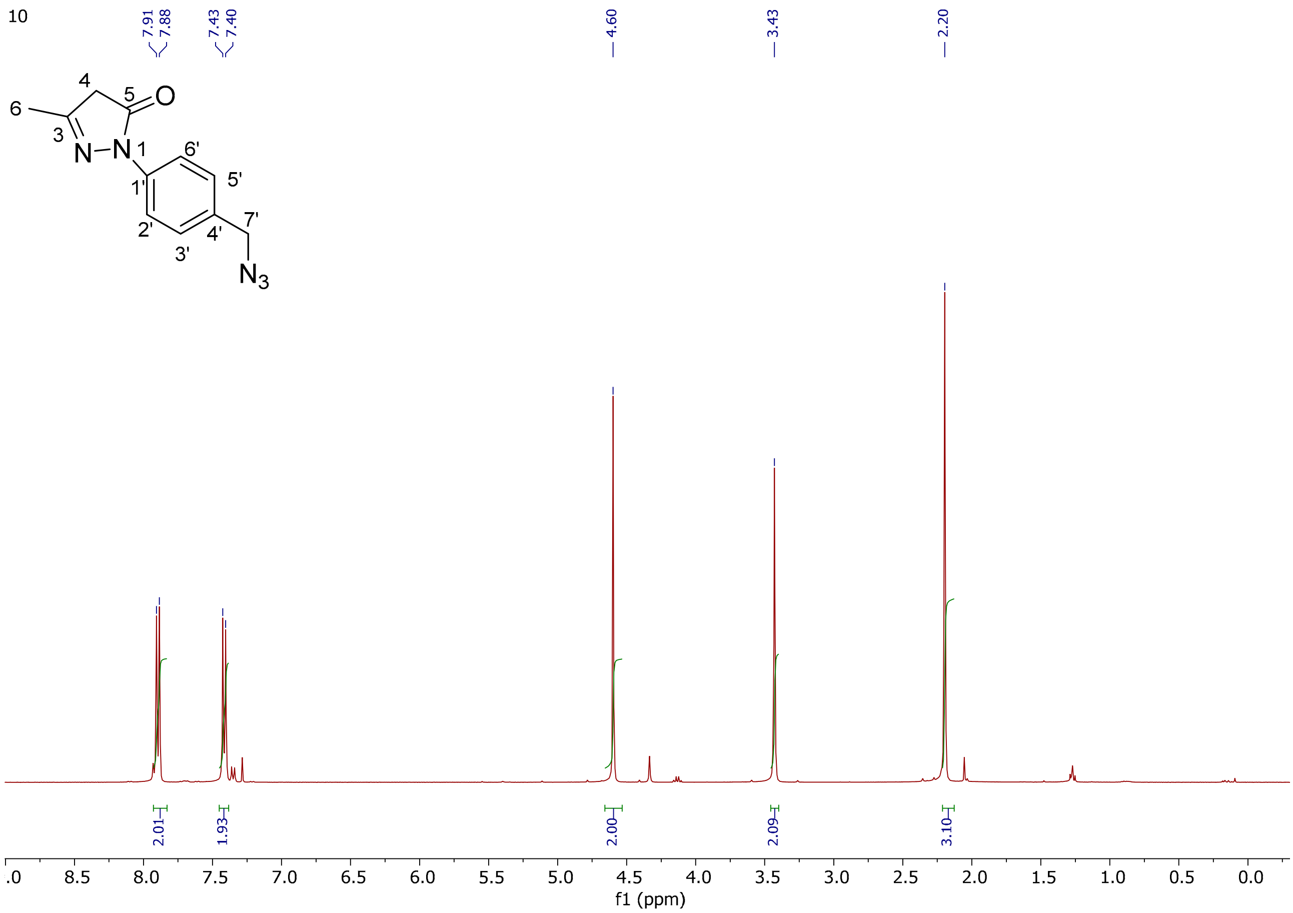

¹H-NMR (400 MHz, $CDCl_3$): δ(ppm) 7.90 (d, 2H, *J* = 8.6 Hz, **H3'-H5'**), 7.42 (d, 2H, *J* = 8.6 Hz, **H2'-H6'**), 4.60 (s, 2H, **H7**), 3.43 (s, 2H, **H4**), 2.20 (s, 3H, **H5**).

10

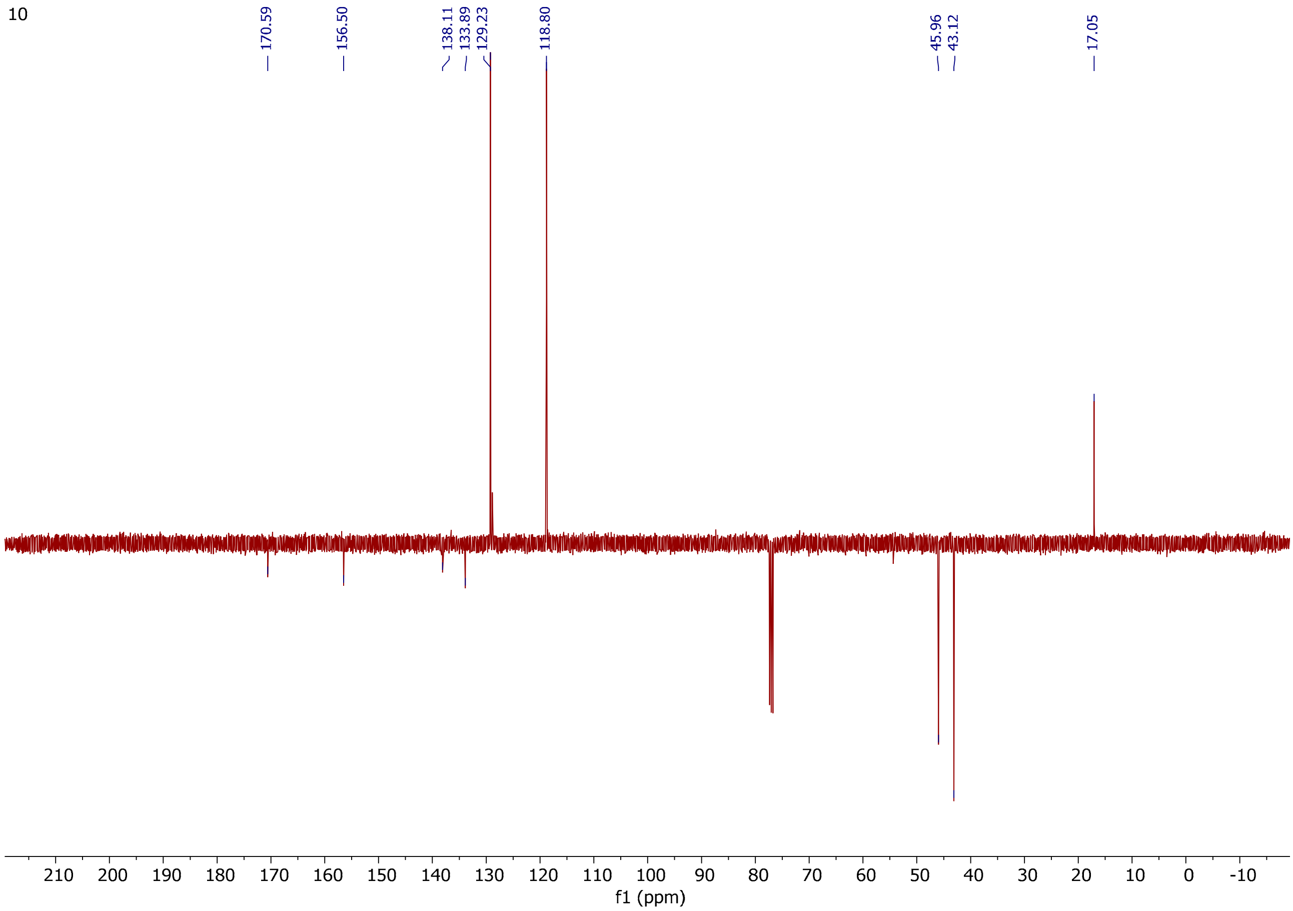

^{13}C-NMR (101 MHz, $CDCl_3$): δ(ppm) 170.6, 156.5, 138.1, 133.9, 129.2 (2C), 118.8 (2C), 46.0, 43.1, 17.0.

MS (ESI), m/z: calcd for $C_{11}H_{11}N_5O$ MW 229.10, found 230.29 ($M+H^+$).

Synthesis of ethyl 4-(benzyloxy)-3-oxobutanoate 5a

60% NaH in mineral oil (801 mg, 39.4 mmol) was washed with n-pentane (20 mL x 3) under nitrogen atmosphere to remove the oil. Then, dry THF (10 mL) was added, and the suspension was stirred at 0°C. Benzyl alcohol (2.05 mL, 19.7 mmol) was added dropwise, and the reaction mixture was stirred at rt for 30 minutes. Then, a solution of ethyl 4-chloroacetoacetate (1.35 mL, 14.7 mmol) in dry THF (10 mL) was added at 0°C, and stirring was continued at 0°C for 30 minutes and at rt for 3 h. The reaction was monitored by TLC (eluent mixture: 98:2 CH_2Cl_2/MeOH) until disappearance of benzyl alcohol. Then, the reaction mixture was diluted with 1M HCl (10 mL), and the aqueous phase was extracted with AcOEt (3 x 25 mL). The collected organic extracts were dried with Na_2SO_4 and evaporated under reduced pressure to a crude which was purified with flash chromatography (silicagel, eluent mixture: 98:2 CH_2Cl_2/MeOH). Pure title compound **5a** was obtained as a brown liquid (3.43 g, 15.6 mmol, 79% yield).

Analytical characterization

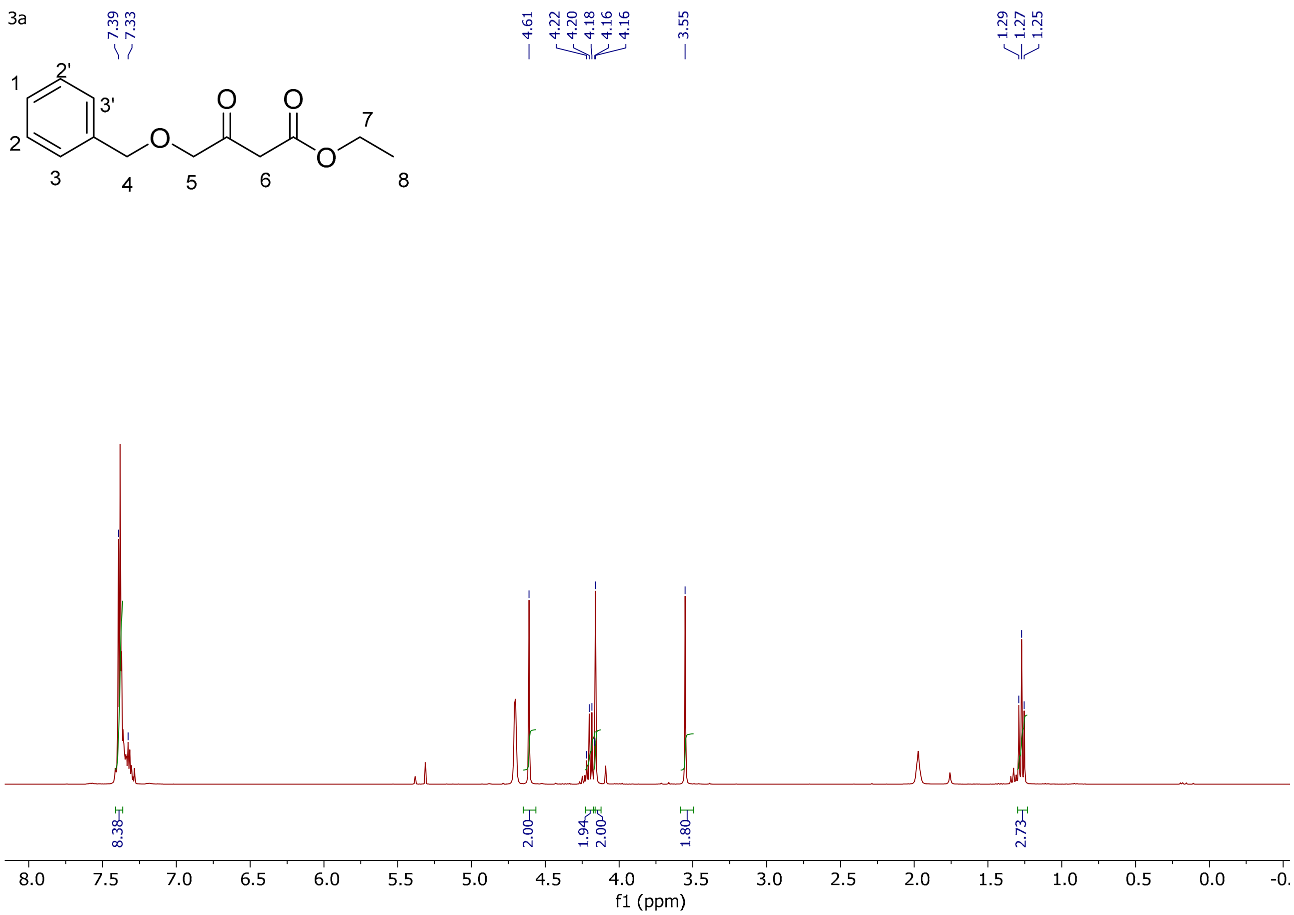

^{1}H-NMR (400 MHz, $CDCl_3$): δ(ppm) 7.39 – 7.33 (m, 5H, **HAr**), 4.61 (s, 2H, **H4**), 4.19 (q, 2H, *J* = 7.5 Hz, **H7**), 4.16 (s, 2H, **H5**), 3.55 (s, 2H, **H6**), 1.27 (t, 3H, *J* = 7.2 Hz, **H8**).

^{13}C-NMR: in accordance with literature data.[3]

MS (ESI), m/z: calcd for $C_{13}H_{16}O_4$ MW 236.11, found 237.41 ($M+H^+$).

Synthesis of 5-((benzyloxy)methyl)-2-phenyl-2,4-dihydro-3H-pyrazol-3-one 3a

Phenylhydrazine (88 L, 0.846 mmol) was added dropwise to a stirred solution of oxobutanoate **5a** (200 mg, 0.846 mmol) in AcOH (4 mL) under nitrogen atmosphere. The reaction mixture was stirred at reflux for 8 h, while monitoring the reaction with TLC (eluent mixture 8:2 n-hex/AcOEt) until disappearance of phenylhydrazine. Then, AcOH was stripped under reduced pressure, and the resulting solid was dissolved in CH_2Cl_2 (5 mL). The solution was washed with saturated aq. NH_4Cl (5 mL). The aqueous phase was extracted with CH_2Cl_2 (3 x 10 mL). The collected organic etracts were dried with Na_2SO_4 and evaporated under reduced pressure to a crude which was purified with flash chromatography (silicagel, eluent mixture 8:2 n-hex/AcOEt). Pure title compound **3a** was obtained as a brown solid (216 mg, 0.639 mmol, 71% yield).

Analytical characterization

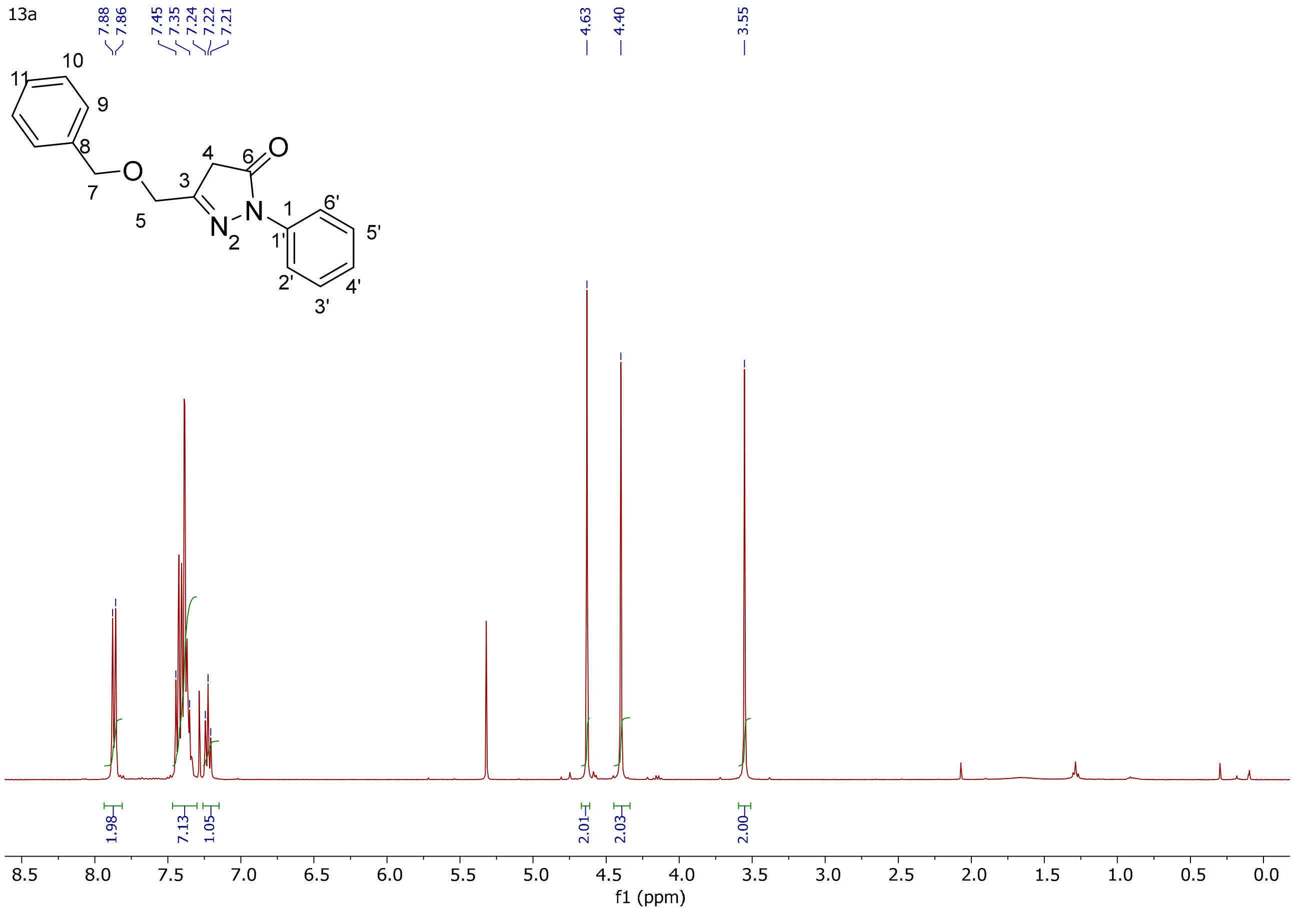

^{1}H-NMR (400 MHz, $CDCl_3$): δ (ppm) 7.86 (d, 2H, *J* = 7.8 Hz, **H3'-H5'**), 7.53 – 7.31 (m, 7H, **H2'-H6'-H9-H9'-H10-H10'-H11**), 7.22 (t, 1H, *J* = 7.4 Hz, **H4'**), 4.63 (s, 2H, **H7**), 4.40 (s, 2H, **H5**), 3.55 (s, 2H, **H4**).

13a

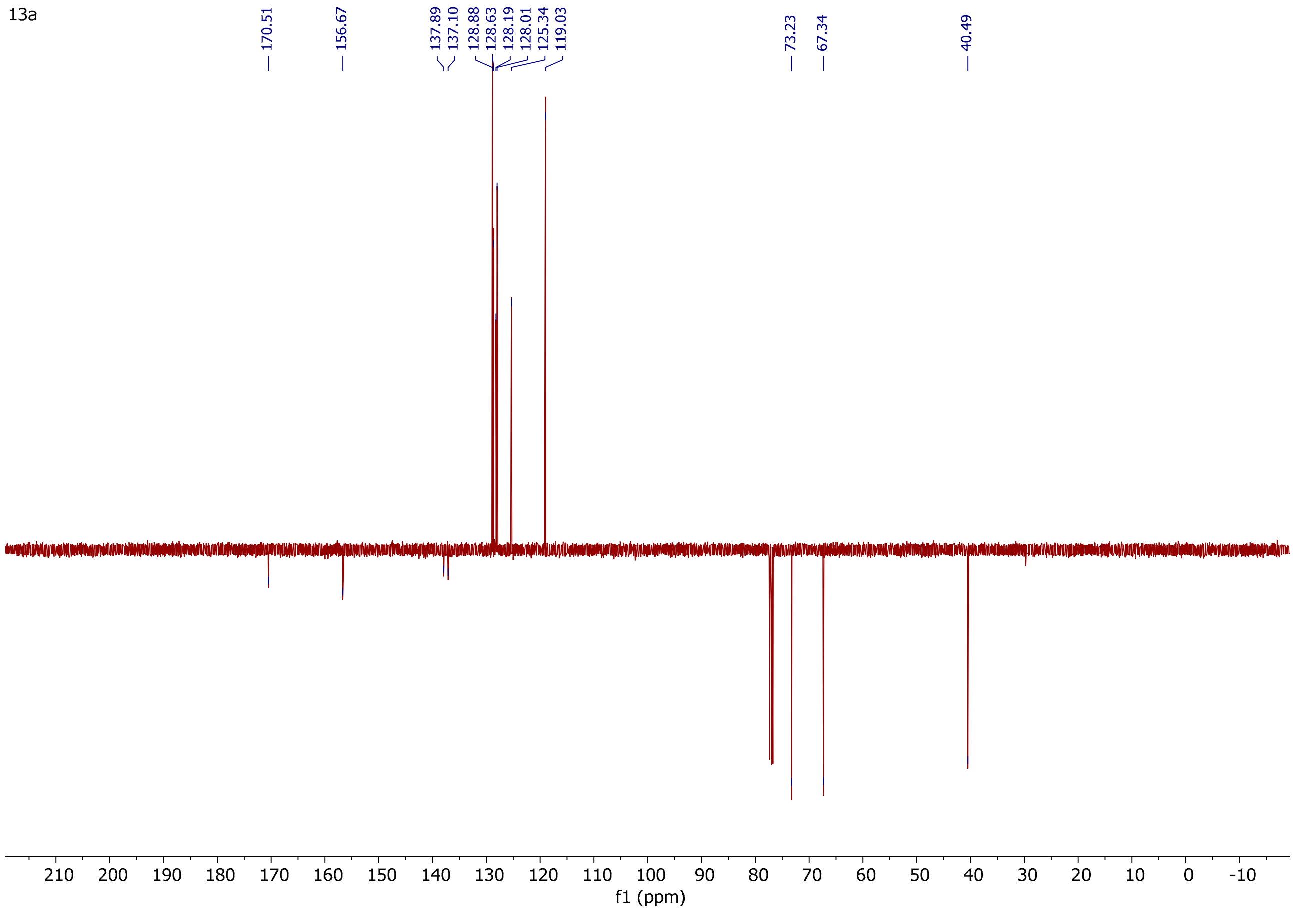

^{13}C-NMR (101 MHz, $CDCl_3$): δ (ppm) 170.5, 156.7, 137.9, 137.1, 128.9 (2C), 128.6 (2C), 128.2 (2C), 128.0, 125.3, 119.0 (2C), 73.2, 67.3, 40.5.

MS (ESI), m/z: calcd for $C_{17}H_{16}N_2O_2$ MW 280.12, found 281.20 ($M+H^+$).

Synthesis of 5-(hydroxymethyl)-2-phenyl-2,4-dihydro-3H-pyrazol-3-one 3b

Pd/C (10% w/w, 107 mg) was added to a solution of **3a** (107 mg, 0.381 mmol) in EtOH (1 mL) under hydrogen atmosphere. The reaction mixture was stirred at rt overnight and was monitored with TLC (eluent mixture 75:25 n-hex/ AcOEt) until disappearance of **3a**. The reaction mixture was then filtered on a celite pad, which was washed with EtOH (5 mL). The organic phase was dried with Na_2SO_4 and evaporated under reduced pressure to a crude which was purified with flash chromatography (silicagel, eluent mixture 75:25 n-hex/AcOEt). Pure title compound **3b** was obtained as a white solid (58.3 mg, 0.301 mmol, 96% yield).

Analytical characterization

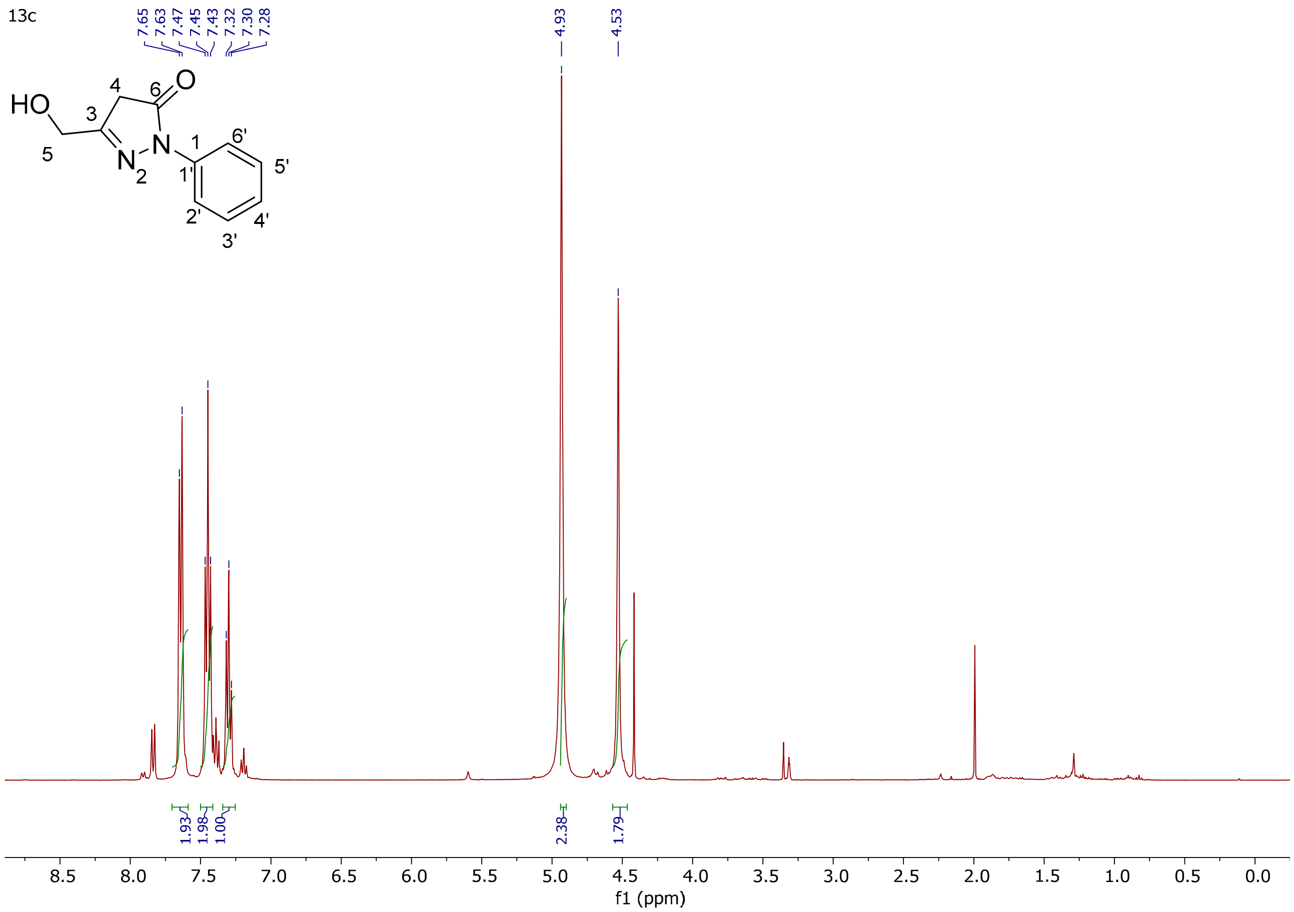

^{1}H-NMR (400 MHz, CD_3OD): δ(ppm) 7.64 (d, 2H, *J* = 7.7 Hz, **H2'-H6'**), 7.45 (t, 2H, *J* = 7.7 Hz, **H3'-H5'**), 7.30 (t, 1H, *J* = 7.2 Hz, **H4'**), 4.93 (s, 2H, **H5**), 4.53 (s, 2H, **H2**).

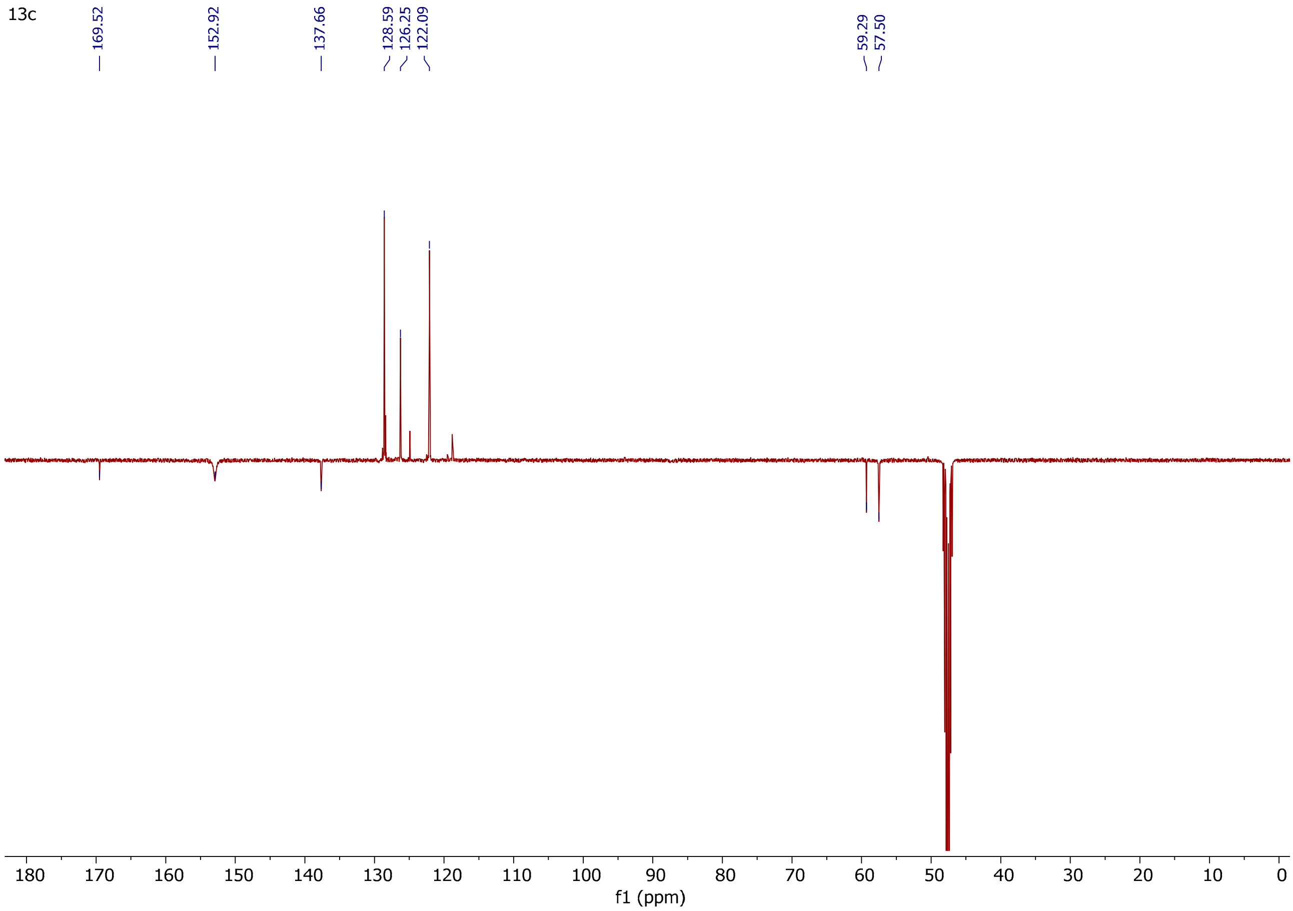

^{13}C-NMR (101 MHz, CD_3OD): δ (ppm) 169.5, 152.9, 137.7, 128.6 (2C), 126.2, 122.1 (2C), 59.3, 57.5.

MS (ESI), m/z: calcd for $C_{10}H_{10}N_2O_2$ MW 190.07, found 191.06 ($M+H^+$).

Chapter 2: Computational Studies

2.1 MEP and ALIE calculations

MEP, as a quantum molecular descriptor, is used to identify molecular sites prone to electrophilic reactions based on charge distribution, and molecular sites prone to nucleophilic attack; conversely, the ALIE descriptor indicates the energy required for the removal of an electron from a given point on a molecule.
MEP and ALIE surfaces obtained by mapping their values onto the electron density surface are shown in Figure S1.

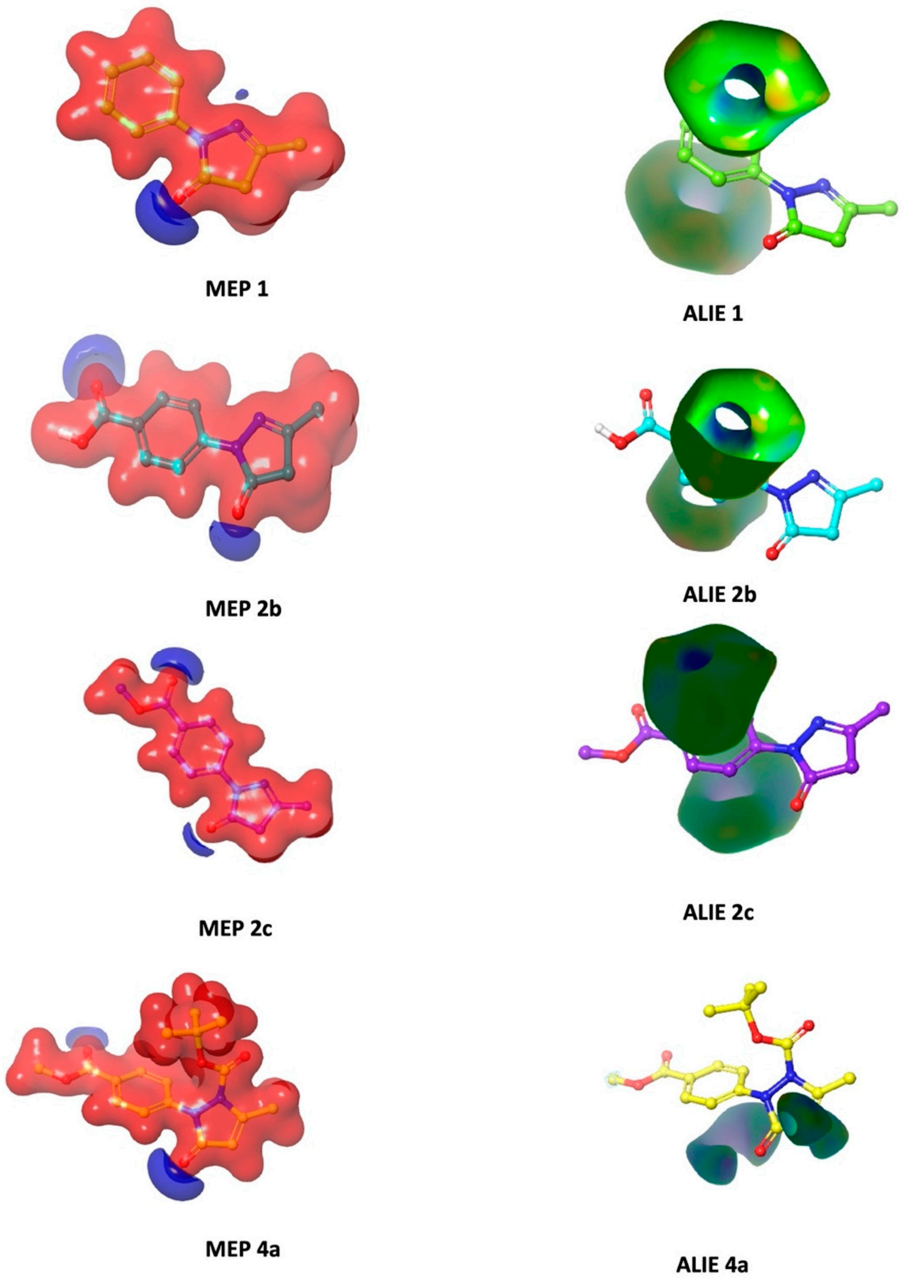

Figure S1 MEP (left) and ALIE plots (right) for EDA **1** (green), **2a** (cyan), **2b** (purple) and **4b** in yellow using DFT analysis.

On the left, MEP plots show negative charges colored in red, corresponding to electrophilic sites, which cover large surface areas; the blue positive areas and correspond to nucleophilic sites located on the carbonyl group in the pyrazole ring, with the exception of carbonyl-free enol ether **6a** (Figure S4, left). As to ALIE on the right, EDA **1**, 4'-carboxy EDA **2b** and 4'-ester EDA **2c** share a most prospective region for electron removal on the phenyl ring, while N^1-carbamate 4'-ester EDA **4b** shows the pyrazolone ring as more prospective. MEP and ALIE exact values are also provided in the Supplementary Information (Table S2).

Table S2. Predicted min-max-average MEP and ALIE values for selected EDA analogues.

**Compound**	**MEP**			**ALIE**		
	min	*max*	*average*	*min*	*max*	*average*
**1**	8.99	14.52	11.58	-1.82	1.42	0.01
**2b**	9.22	15.94	11.86	-1.90	2.16	0.03
**2c**	9.20	14.93	11.82	-1.98	1.60	-0.01
**4b**	8.87	14.94	11.81	-2.28	1.07	0.04

2.2 HOMO and LUMO calculations

The HOMO (Highest Occupied Molecular Orbitals) and LUMO (Lowest Unoccupied Molecular Orbitals) representation, and the ΔE energy gap for EDA analogues are shown as images in Figure S2.

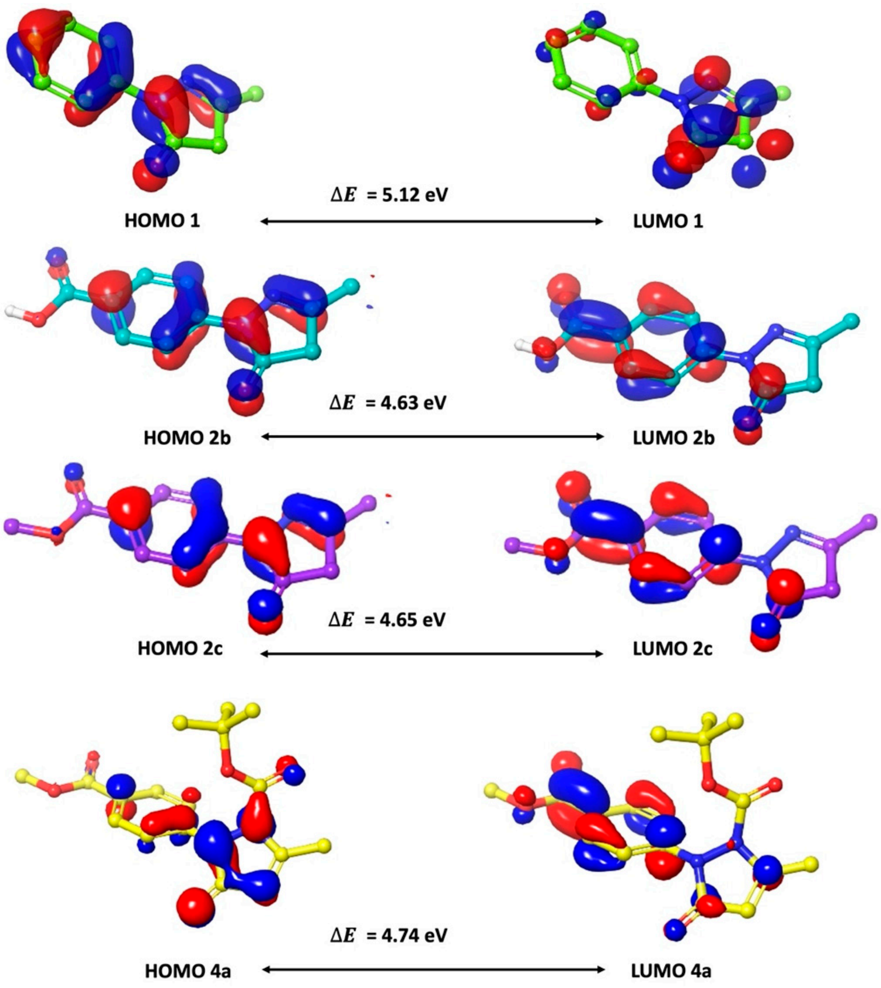

Figure S2: Atomic HOMO - LUMO orbital representation and E energy gap values for (top to bottom) EDA **1** (green), 4'-carboxy EDA **2b** (cyan), 4'-ester EDA **2c** (purple) and N^{1}-carbamate 4'-ester EDA **4b** (yellow) using DFT analysis.

REFERENCES

(1) Holzer, W.; Plagens, B.; Lorenz, K. Alkylation of Pyrazolones via the Mitsunobu Reaction. *Heterocycles* **1997**, *45*, 309–314.

(2) Haessner, R.; Hennig, L.; Gaca, J. 13C NMR Data for Chlorine- or Nitro-Substituted Azomethine Dyes. *Magn. Reson. Chem.* **1990**, *28*, 817–819.

(3) Fan, W. et al. Ru-Catalyzed Asymmetric Hydrogenation of γ-Heteroatom Substituted β-Keto Esters. *J. Org. Chem.* **2011**, *76*, 9444–9451.

(4) Kotha, S.; Shirbhate, M. E. Diversity-Oriented Approach to Macrocyclic Cyclophane Derivatives via Ring-Closing Metathesis. *Synlett* **2012**, *23*, 2183–2188.
